# Machine learning-enabled chemical ecology for integrated pest management: from volatiles to field applications

**DOI:** 10.1093/jisesa/ieag087

**Published:** 2026-09-02

**Authors:** Steve B S Baleba, Victor O Omondi, Pascal Aigbedion-Atalor, Emmanuel Peter, Souleymane Diallo, Komi Mensah Agboka

**Affiliations:** Behavioural and Chemical Ecology Unit, International Centre of Insect Physiology and Ecology (icipe), Duduville, Kasarani, Nairobi, 30772-00100, Kenya; Department of Zoology and Entomology, University of Pretoria, Hatfield, South Africa; Behavioural and Chemical Ecology Unit, International Centre of Insect Physiology and Ecology (icipe), Duduville, Kasarani, Nairobi, 30772-00100, Kenya; Department of Zoology and Entomology, University of Pretoria, Hatfield, South Africa; Department of Plant and Environmental Protection Sciences, College of Tropical Agriculture and Human Resilience, University of Hawaii at Manoa, Honolulu, HI, USA; College of Tropical Agriculture and Human, Resilience, University of Hawaii at Manoa, Komohana Center for Applied Research and Extension Services, Hilo, HI, USA; Behavioural and Chemical Ecology Unit, International Centre of Insect Physiology and Ecology (icipe), Duduville, Kasarani, Nairobi, 30772-00100, Kenya; Faculty of Agriculture, Federal University Gashua, Gashua, Yobe State, Nigeria; Behavioural and Chemical Ecology Unit, International Centre of Insect Physiology and Ecology (icipe), Duduville, Kasarani, Nairobi, 30772-00100, Kenya; Behavioural and Chemical Ecology Unit, International Centre of Insect Physiology and Ecology (icipe), Duduville, Kasarani, Nairobi, 30772-00100, Kenya; Laboratoire de Recherche en Science et Technologie (LARSI), Département de Génie Informatique (GI), École Polytechnique de Lomé (EPL), Université de Lomé, Lomé, Togo

**Keywords:** Ecology, Semiochemical, Behaviour, Pests, Management, Algorithms

## Abstract

Machine learning is transforming chemical ecology by accelerating the discovery and deployment of semiochemical-based tools for precision pest management. These advances are particularly important in the face of climate change, pesticide resistance, and the growing need for sustainable agricultural intensification. This review synthesizes how machine learning can be applied across the semiochemical discovery and implementation pipeline, from chemical signal detection to field deployment and decision support for integrated pest management. We review major machine learning approaches and demonstrate how they extract biologically relevant information from high-dimensional chemical, electrophysiological, behavioral, sensor, and field datasets. These methods accelerate semiochemical discovery, prioritize candidate compounds, optimize formulations and deployment strategies, and support adaptive pest management under dynamic environmental conditions. We further examine the integration of sensor-based technologies, explainable machine learning, and optimization algorithms to improve pest detection, monitoring, and intervention. Current challenges include data scarcity, heterogeneous datasets, the lack of benchmark resources, limited field validation, and the underrepresentation of tropical agroecosystems and smallholder farming systems. We propose practical recommendations for developing reusable datasets that integrate volatile profiles, electrophysiology, insect behavior, and management outcomes to improve model robustness and reproducibility. Central to this review is a 4-stage framework comprising detection, design, deployment, and decision support that connects machine learning with chemical ecology and provides a practical roadmap for translating computational advances into targeted, efficient, and sustainable pest management.

## Introduction

Effective pest management increasingly relies on understanding how insects detect and respond to chemical cues, particularly under growing pressures from climate change, pesticide resistance, and the need for sustainable intensification. Chemical ecology provides the mechanistic foundation for this understanding, supporting behaviorally based pest management strategies (Allan 2018, Mbaluto et al. 2020, Gaffke et al. 2021). Semiochemicals, classified into pheromones and allelochemicals (Abd El-Ghany 2023, Baleba 2026), underpin key pest management strategies, including monitoring, mating disruption, mass trapping, biological control, attract and kill systems, and crop diversification practices such as push–pull and intercropping, which enhance agroecosystem resilience and reduce pest pressure (Guerrero and Reddy 2023, Guo and Wang 2025). In many systems, behavioral responses depend not on individual compounds but on combinations of cues from different sources, including sex and aggregation pheromones, host plant volatiles, herbivore-induced plant volatiles, and food odors (Reddy and Guerrero 2004, Webster and Cardé 2017). Behaviorally relevant signals are therefore rarely single compounds. Rather, they are dynamic, context-dependent mixtures influenced by chemical composition, release patterns, insect physiology, plant phenology, and environmental conditions (Aflitto et al. 2023, Serdo 2024, Thompson et al. 2026). Data generated from Gas Chromatography Mass Spectrometry (GC-MS), electrophysiology, behavioral assays, field trapping, and sensor networks are often high-dimensional and heterogeneous, posing challenges to conventional statistical methods designed for low-dimensional, hypothesis-driven analyses (Hervé et al. 2018, Barbosa-Cornelio et al. 2019).

These challenges have created a need for analytical approaches capable of handling complex, high-dimensional datasets beyond the scope of traditional statistical methods. Data-driven approaches, particularly those capable of capturing nonlinear relationships and integrating heterogeneous data sources, are therefore increasingly required. In this context, advances in data science and artificial intelligence (AI) are transforming pest management. Machine learning (ML) integrates diverse data streams, enabling pest detection, forecasting, and decision support across cropping systems (Domingues et al. 2022, Amiri and Bandan 2026, Botero-Valencia et al. 2025, Shoaib et al. 2025). In chemical ecology, ML is particularly valuable for learning nonlinear relationships, detecting hidden structures, and generalizing across contexts. Models now predict volatile–protein interactions, prioritize semiochemical candidates, and classify odor-driven electrophysiological signals. Different ML algorithms are suited to different analytical tasks. For example, tree-based boosting algorithms, such as XGBoost and LightGBM, sequentially combine multiple decision trees to improve predictive accuracy and are particularly effective for analyzing structured datasets with complex nonlinear relationships. These models have been used to accurately predict volatile binding to odorant-binding proteins, facilitating targeted screening (López-Cortés et al. 2025). Similarly, support vector machines (SVMs), which identify the optimal boundary separating different classes, and random forests (RFs), which combine predictions from multiple decision trees to improve robustness and reduce overfitting, have been applied to classify odor-derived electroantennogram responses in the tobacco hawkmoth *Manduca sexta* (Linnaeus) (Lepidoptera: Sphingidae), thereby automating stimulus discrimination (Swore et al. 2025). These applications address 2 long-standing challenges: identifying biologically relevant compounds in complex blends and interpreting high-dimensional physiological signals.

Despite these advances, most studies remain task-specific, focusing on ligand prediction, signal classification, or image-based detection, without integration into operational Insect Pest Management (IPM) decision-making frameworks. As a result, ML applications in chemical ecology remain fragmented. For example, optimization approaches for trap networks and spatial monitoring are often disconnected from upstream semiochemical discovery (Duan et al. 2024, Yanchenko et al. 2025). Similarly, sensor-based early warning systems typically treat pest presence as a detection problem, underutilizing chemical cues that could improve forecasting (Kim and Sim 2025). This fragmentation is further reflected in geographical and production system biases. For instance, most ML-driven studies target well-resourced temperate systems, while tropical and smallholder agroecosystems, with high pest pressure and limited chemical inputs, remain underrepresented. Initiatives such as AI-driven palm weevil monitoring in Ghana (Innovate UK 2025) demonstrate feasibility but highlight the need for low-cost, scalable solutions adapted to these contexts.

**Fig. 1.**
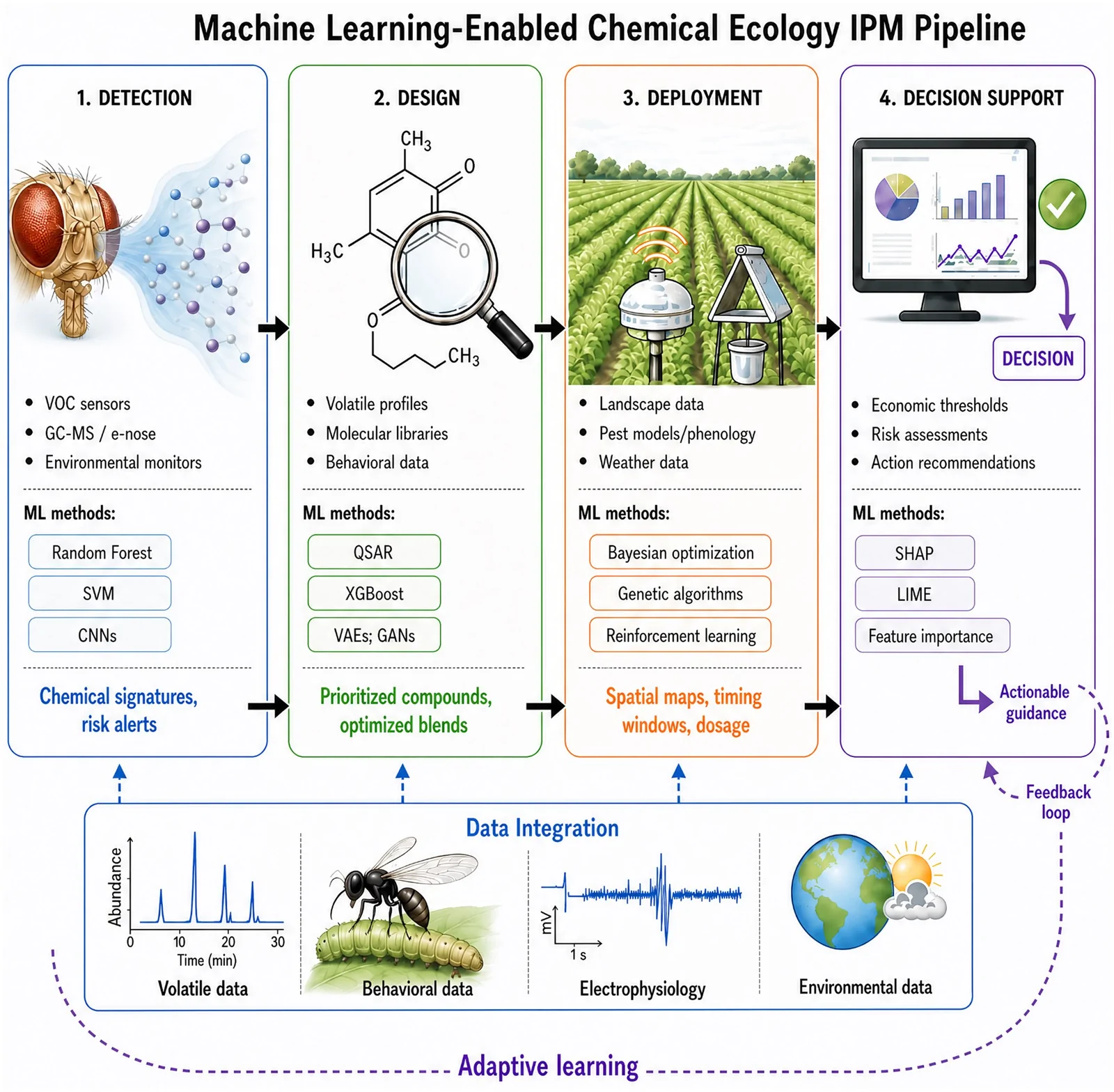
ML-enhanced IPM pipeline integrating chemical ecology data across 4 stages with adaptive feedback. “Stage 1 (Detection)”: VOC sensors, GC-MS/e-nose, and environmental monitors feed Random Forest, SVM, and CNNs to generate chemical signatures and risk alerts. Olfactory sensing illustrated by a fly head with odor molecules approaching antennae. “Stage 2 (Design)”: Volatile profiles, molecular libraries, and behavioral data inform QSAR, XGBoost, VAEs, and GANs for compound prioritization and blend optimization (molecule with magnifying glass). “Stage 3 (Deployment)”: Landscape data, pest phenology, and weather data guide Bayesian optimization, genetic algorithms, and reinforcement learning to produce spatial maps, timing windows, and dosages (crop rows with dispenser and trap). “Stage 4 (Decision Support)”: Economic thresholds, risk assessments, and action recommendations processed through SHAP, LIME, and feature importance deliver actionable guidance. Dashed feedback loop (stage 4 → stage 1) enables adaptive learning. “Data Integration Layer” (bottom) synthesizes volatile, behavioral, electrophysiological, and environmental data via vertical connections to all stages.

Collectively, these gaps highlight the need for a more integrated framework. Although recent developments suggest that ML can connect chemical cue discovery, behavioral interpretation, spatial optimization, and real-time decision support (Baleba et al. 2019, Kariyanna and Sowjanya 2024, Orubuloye et al. 2025), the field still lacks a coherent synthesis that organizes these advances into a structured IPM pipeline. Without such a framework, researchers and practitioners struggle to select appropriate ML tools, integrate diverse data streams, or identify key limitations. This review, therefore, provides a comprehensive synthesis of ML applications in chemical ecology for pest management, linking data science to applied entomology. The methods, applications, challenges, and opportunities discussed throughout this review are intended as representative examples rather than an exhaustive catalogue of the rapidly expanding literature. We cover supervised, unsupervised, generative, and optimization-based methods across the semiochemical pipeline—from signal discovery to operational decision support. We first examine ML for the deconvolution of complex volatile signatures and electrophysiological signals. Next, we elucidate the role of predictive and generative models for discovering and optimizing attractants and repellents and then evaluate spatial and temporal optimization for semiochemical deployment, drawing on trap network design and links to landscape structure, crop phenology, and pest dynamics. Emerging sensor-based early warning systems are discussed, with emphasis on incorporating chemical cues. Also, we consider explainable ML for decision support, linking chemical signals, environmental factors, and crop development stages to actionable recommendations. Finally, we discuss key challenges, including data scarcity, heterogeneous data integration, the lack of shared benchmarks, and limited field validation, and provide practical guidance for developing reusable datasets. By framing ML across 4 operational IPM stages—detection, design, deployment, and decision support (Fig. 1)—this review presents an applied framework for translating chemical ecology insights into operational, scalable, and context-appropriate pest management tools.

## Literature Search and Review Approach

We conducted a narrative review to synthesize evidence on ML applications in chemical ecology for integrated pest management. Our objectives were to identify current applications, highlight knowledge gaps, and develop a framework that links ML to semiochemical-based pest management.

We searched Google Scholar, Web of Science, Scopus, PubMed, and CAB Abstracts between January and May 2026. We updated the searches immediately before manuscript submission to capture recently published studies. Search terms combined ML concepts, including AI, deep learning, RF, SVM, XGBoost, LightGBM, neural networks, explainable AI, Bayesian optimization, and generative models, with chemical ecology terms such as semiochemicals, pheromones, volatile organic compounds (VOCs), insect olfaction, electrophysiology, GC-MS, behavioral assays, and integrated pest management. We also conducted targeted searches on volatile discovery, semiochemical blend optimization, olfactory receptor prediction, sensor-based pest detection, trap deployment, and decision support systems (DSS). We identified additional studies from the reference lists of retrieved articles and from our knowledge of the field. We included studies that applied ML to chemical ecology data, semiochemical discovery or optimization, insect behavioral or electrophysiological datasets, chemical analyses, sensor-based monitoring, or operational pest management. We also included studies reporting laboratory or field validation of machine-learning-guided approaches. We excluded studies unrelated to chemical ecology or integrated pest management, including image-based pest detection without chemical ecology components, studies on noninsect systems, and studies unrelated to volatile- or semiochemical-mediated interactions.

We imposed no publication date restriction. We included older studies that established foundational concepts in chemical ecology, insect olfaction, structure odor relationships, and semiochemical-based pest management. We emphasized studies published since 2000 because they reflect the rapid development of ML and its application to biological data. We prioritized studies supported by electrophysiological, behavioral, or field validation whenever possible. For each study, we extracted information on (i) the ML methods, (ii) chemical ecology data, (iii) biological system, (iv) validation approach, principal findings, model performance where relevant, and (v) reported limitations. We also recorded each study’s contribution to semiochemical discovery, blend optimization, deployment strategies, monitoring systems, and decision support. We synthesized the literature by comparing methods, applications, and biological outcomes across studies. Based on this synthesis, we developed a 4-stage framework comprising detection, design, deployment, and decision support, which forms the structure of this review. We distinguish among established applications supported by experimental evidence, emerging approaches with proof-of-concept validation, and promising directions that remain to be tested. Following recommended practice for narrative reviews, we describe our search strategy, selection criteria, and evidence synthesis to maximize transparency while recognizing that this review is a critical narrative synthesis rather than a systematic review (Green et al. 2006, Torraco 2016, Snyder 2019).

## Foundations of Chemical Ecology Data and ML Integration

### The Nature of Chemical Ecology Data: High-Dimensionality, Heterogeneity, and Complexity

Chemical ecology has its roots in early studies of chemically mediated interactions among plants, insects, and other organisms, and became established as a distinct scientific discipline during the mid-twentieth century through major advances in identifying biologically active natural compounds, including insect pheromones (Karlson and Lüscher 1959). Since then, the field has evolved from describing individual semiochemicals to understanding complex multimodal signaling networks and now integrates molecular, neurobiological, ecological, and computational approaches to investigate how chemical signals mediate ecological and evolutionary processes across multiple levels of biological organization. Chemical ecology research is fundamentally behavior-driven (Aigbedion-Atalor et al. 2024a,b, Baleba et al. 2026), as behavioral responses ultimately define the biological relevance of chemical signals and determine whether they can be translated into effective pest management interventions. Consequently, chemical ecology datasets are inherently multimodal and behavior-anchored, linking behavioral responses with chemical profiles, sensory processing, and environmental context.

#### Chemical Data and Blend Representations

**Fig. 2.**
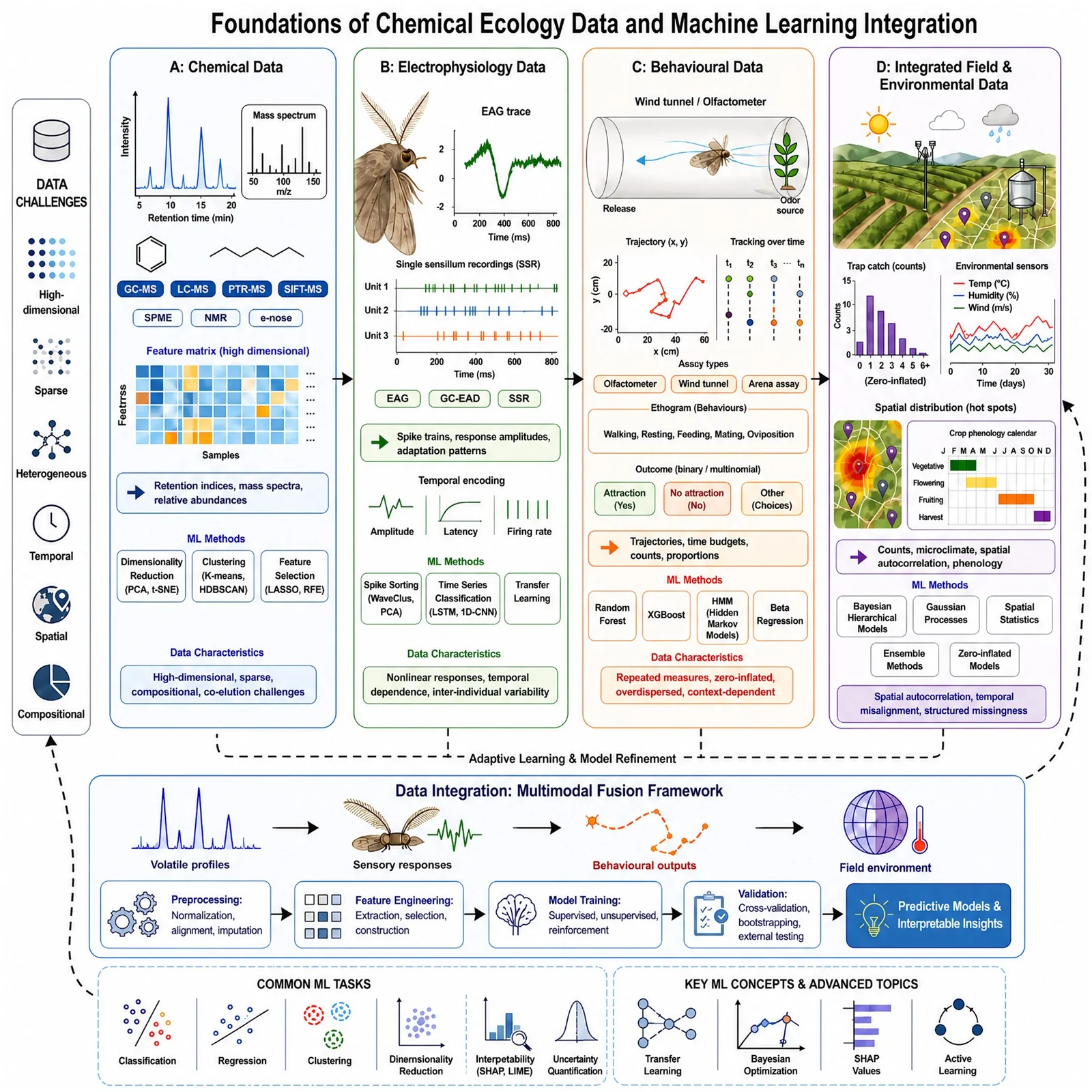
Multimodal data landscape in chemical ecology and corresponding machine learning (ML) approaches across 4 interconnected data modalities. “A) Chemical Data”: High-dimensional volatile profiles from GC-MS, LC-MS, and real-time MS techniques (PTR-MS, SIFT-MS), complemented by NMR, SPME, and e-nose. Data are compositional, sparse, and affected by co-elution and annotation challenges. ML methods include dimensionality reduction (PCA, *t*-SNE), clustering (k-means, HDBSCAN), and feature selection (LASSO, RFE). “B) Electrophysiology Data”: EAG, GC-EAD, and SSR recordings capture olfactory receptor activity through spike trains and temporal response patterns (amplitude, latency, firing rate). Data exhibit nonlinearity, temporal dependence, and inter-individual variability. ML approaches: spike sorting, time-series classification (LSTM, 1D-CNN), and transfer learning. “C) Behavioral Data”: Olfactometer, wind tunnel, arena, and field assays generate trajectories, time budgets, and count data with repeated measures and zero-inflation. ML methods: Random Forest, XGBoost, HMMs, and Beta regression for compositional outcomes. “D) Integrated Field and Environmental Data”: Trap catches, microclimate sensors, and spatial data with autocorrelation, temporal misalignment, and structured missingness. ML approaches: Bayesian hierarchical models, Gaussian processes, and zero-inflated models. “Data Integration Layer” (bottom): Fuses volatile profiles, sensory responses, behavioral outputs, and field environmental data through preprocessing, feature engineering, model training, and validation pipelines. Dashed feedback loop (D → A) enables adaptive learning and continuous model refinement. Common ML tasks (classification, regression, clustering, dimensionality reduction) and interpretability tools (SHAP, LIME) support predictive modeling and mechanistic understanding across all data types.

Chemical ecology datasets, primarily generated using GC-MS, provide high-resolution volatile profiles (Hayes et al. 2023) (Fig. 2A). Single samples may contain hundreds of compounds, represented as feature matrices comprising retention indices, mass spectra, and relative peak abundances. These datasets are typically high-dimensional and sparse, and are often affected by missing peaks, peak alignment errors, and correlated variables. Liquid chromatography mass spectrometry (LC-MS) captures polar metabolites (Alanazi 2025, Liao et al. 2025), while Proton Transfer Reaction mass spectrometry (PTR-MS) and Selected Ion Flow Tube mass spectrometry (SIFT-MS) enable real-time monitoring of volatile emissions (Liu et al. 2018, Majchrzak et al. 2018). Complementary methods, including Solid Phase Microextraction, direct injection MS, Nuclear Magnetic Resonance spectroscopy, sensor arrays, and electronic noses, expand chemical coverage and facilitate rapid monitoring (Emwas et al. 2019, Karakaya et al. 2020, Rabehi et al. 2024, Leni et al. 2025). Large-scale curation highlights additional sources of heterogeneity, as compound annotations vary across databases and reference spectra differ in quality and completeness (Schulz and Möllerke 2022). Large-scale curation highlights additional sources of heterogeneity, as compound annotations vary across databases and reference spectra differ in quality and completeness (Schulz and Möllerke 2022). Compound annotation and identification may also be affected by coeluting peaks, ambiguous spectral matches, retention index variation, and incomplete reference libraries. These sources of uncertainty can propagate through downstream analyses, influencing feature selection, model development, and biological interpretation if they are not adequately addressed. Chemical data are compositional because relative abundances are constrained and influenced by sampling artifacts. Biological activity typically arises from mixtures rather than individual compounds. Behavioral responses depend on blend composition, relative proportions, and interactions among components (Tasin et al. 2007, Piñero and Dorn 2007, Piñero et al. 2008, Varela et al. 2011). Temporal dynamics further increase complexity because emissions change with plant phenology, herbivore damage, and environmental conditions (Faiola and Taipale 2020, Hamann et al. 2021). Chemical datasets, therefore, represent dynamic, context-dependent descriptions of biologically relevant signals.

#### Electrophysiological Data and Temporal Encoding

Electrophysiological recordings link chemical stimuli to olfactory receptor activity, revealing which volatile compounds insects can detect before central processing and behavioral responses (Fig. 2B). Electroantennography (EAG) and GC EAD produce continuous time series that reflect the summed receptor potentials of olfactory receptor neurons (Olsson and Hansson 2013, Shuttleworth and Johnson 2022). GC-EAD datasets combine chromatographic retention times with electrophysiological responses, requiring accurate alignment between chemical and neural signals to identify biologically active compounds. Single sensillum recordings (SSRs) generate spike trains from individual olfactory receptor neurons (Den Otter et al. 1980, Baleba et al. 2023). Sensory information is encoded in amplitude, latency, firing patterns, synchrony, and adaptation (Lei et al. 2004, Chong et al. 2012, Stierle et al. 2013, Ling et al. 2025). Temporal structure conveys stimulus identity, concentration, and mixture composition (Riffell et al. 2009, Su et al. 2011). Responses are nonlinear, influenced by prior stimulation, physiological state, and compound interactions (Arenas et al. 2009, Renou and Anton 2020), and exhibit substantial temporal dependence and inter-individual variability, challenging traditional summary-based analyses. ML is particularly well suited to these data because it can model nonlinear response patterns, exploit temporal structure, and classify complex electrophysiological signals without relying on predefined assumptions about the underlying biological relationships (Craik et al. 2019, Vaid et al. 2022, Swore et al. 2025).

#### Behavioral Data and Response Structures

Behavioral assays generate diverse and structurally complex data (Fig. 2C). Olfactometer and wind tunnel experiments produce binary, multinomial, or continuous trajectory data describing attraction, orientation, flight dynamics, and search behavior (Knudsen et al. 2018, Subhash and Shashank 2019, Rodrigues et al. 2022, Van Winkle et al. 2022, Masini et al. 2024). Video tracking systems extend these methods, generating high-resolution time series of position, speed, turning angles, and plume-following dynamics (Van Breugel and Dickinson 2014, Baleba et al. 2024, Vo-Doan et al. 2024). Arena and cage assays produce multivariate time-budget data describing locomotion, resting, feeding, grooming, mating, and egg-laying activities, which are compositional in nature (Rodríguez López et al. 2012, Seehausen et al. 2022, Scharf et al. 2024, Tuñón et al. 2025). Field and semi-field experiments generate count- or event-based data that are often overdispersed, zero-inflated, and temporally structured (Richards 2008, Mugenyi et al. 2021, Pogue et al. 2024). Behavioral responses are influenced by physiological state, age, mating status, nutritional condition, prior experience, and morph (Reese 1996, Arnold et al. 2012, Webster 2012, Fan et al. 2015, Zhang et al. 2019, Rádai et al. 2022, Lenschow et al. 2018, Singh et al. 2023, Wu et al. 2023, Greene et al. 2025). Responses also vary across diel cycles and environmental conditions, creating structured heterogeneity rather than noise (Carter et al. 2025, Tyler et al. 2025). Behavioral variability reflects adaptive plasticity shaped by experience and environmental context (Nussey et al. 2007, Dingemanse and Wolf 2013). This variation is structured within and among individuals rather than random noise (Bell et al. 2009, Hertel et al. 2020). Reliable analysis, therefore, requires frameworks that account for repeated measures, temporal dependence, nonlinear responses, and nonnormal data structures (Stamps 2016, Mitchell et al. 2019).

#### The Data Landscape of Chemical Ecology

Field experiments introduce spatial and temporal dimensions (Fig. 2D). Trap catch data are influenced by landscape structure, crop distribution, pest population dynamics, temperature, humidity, wind, moon phase, crop phenology, and plant physiology (Dent and Pawar 1988, Mitchell 2008, Ricci et al. 2009, Silva et al. 2015, Daems et al. 2016, Karp et al. 2018, Ahmed et al. 2023, M’sakni et al. 2025, Moliterno et al. 2025, Borg et al. 2026). These datasets are frequently sparse, overdispersed, and zero-inflated because many sampling locations or sampling periods record no captures. Continuous sensor deployments generate microclimatic time series and automated observations (Mansoor et al. 2025, Nyakuri et al. 2025, Nramat et al. 2026). Integrating these continuous measurements with discrete behavioral, chemical, and trap catch data produces multiresolution datasets characterized by temporal misalignment, spatial autocorrelation, temporal dependence, and structured missing observations. Integrated studies increasingly combine behavioral trajectories, chemical profiles, electrophysiology, and environmental measurements (Baraki et al. 2025, Pistillo et al. 2025), resulting in heterogeneous, partially observed datasets. Complexity ultimately arises from biological context, where behavioral responses emerge through interactions among blend composition, insect physiological state, and environmental conditions.

#### Limitations of Traditional Statistical Approaches

Classical statistical tools, including ANOVA, linear regression, and multivariate analyses, are indispensable for hypothesis testing, parameter estimation, and quantifying relationships between variables but typically assume normality, independence, and homoscedasticity (Kim 2015, Vandever 2020, Rajić 2026). Nonparametric alternatives, such as the Wilcoxon, Kruskal–Wallis, and Friedman tests, relax some of these assumptions and are well suited to skewed distributions and small sample sizes (Wagner et al. 2011, MacFarland and Yates 2016). However, these approaches generally require predefined hypotheses, rely on investigator-selected variables, and may have limited ability to capture complex interactions within high-dimensional datasets. In chemical ecology, where behavioral activity results from interactions among multiple compounds, dynamic contexts, and nonlinear temporal patterns (Webster et al. 2010, Mbaluto et al. 2020, Patricelli 2023, Niu et al. 2024, Thompson et al. 2026), these characteristics can make it difficult to identify subtle patterns and emergent relationships. Behavioral and chemical variability often contain biologically meaningful information rather than simply representing experimental noise. ML therefore complements, rather than replaces, conventional statistical approaches by providing data-driven methods that can model nonlinear relationships, integrate heterogeneous and high-dimensional datasets, exploit temporal structure, and improve prediction and pattern recognition when these are the primary analytical objectives (Olden et al. 2008, Pichler and Hartig 2023, Yu et al. 2025).

### ML Paradigms in Chemical Ecology: Supervised, Unsupervised, and Generative Approaches

**Fig. 3.**
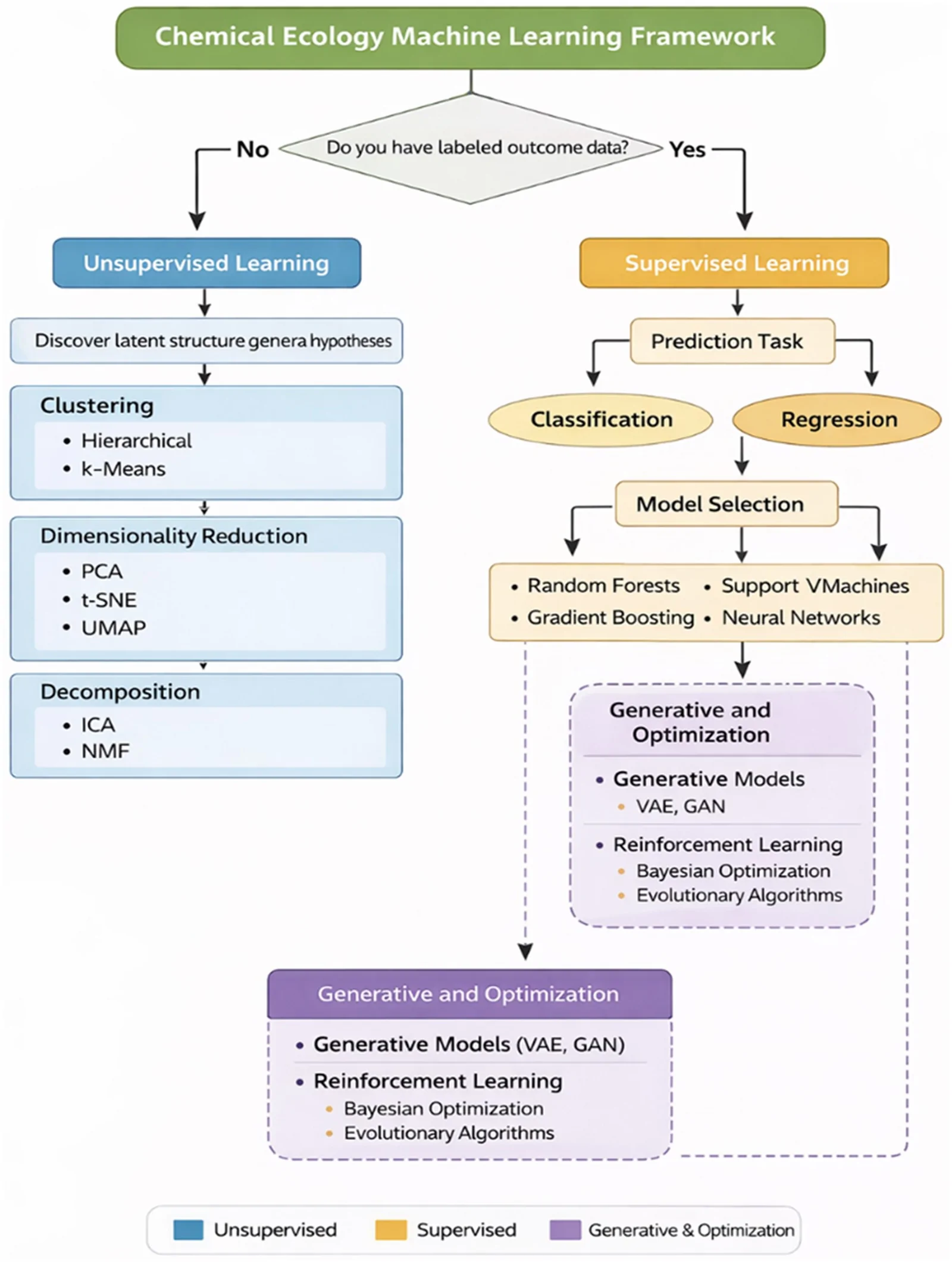
Decision tree for selecting machine learning approaches in chemical ecology based on research objectives, data characteristics, and analytical requirements. Researchers begin by determining whether labeled outcome data (eg behavioral responses, electrophysiological classifications, or compound activity) are available. In the absence of labeled data, the unsupervised learning branch (light blue) guides the selection of methods for dimensionality reduction, clustering, or signal decomposition to explore data structure and generate hypotheses for experimental validation. When labeled data are available, the supervised learning branch (light orange) recommends models according to dataset size, prediction objective (classification or regression), and the need for model interpretability or mechanistic insight. A parallel branch (light purple) highlights generative and optimization-based approaches for molecular design, deployment optimization, and sequential decision-making. The decision tree is intended as a practical guide for selecting an appropriate ML strategy rather than a prescriptive workflow, with final model choice also depending on data quality, sample size, computational resources, and the biological question being addressed. Colours denote the different learning paradigms, and behavioral relevance and experimental validation should guide interpretation of all model outputs.

ML provides analytical tools designed for high-dimensional, heterogeneous, and nonlinear data. These properties match the structure of modern chemical ecology datasets. Four broad paradigms, including (i) unsupervised learning, (ii) supervised learning, (iii) generative learning, and (iv) optimization-based learning, are particularly relevant (Table 1). Each addresses distinct biological questions and data types across the semiochemical discovery-to-deployment pipeline. Figure 3 provides a decision tree to guide method selection based on data characteristics and research goals. Practical implementation of these approaches has become increasingly accessible through open-source software. Most ML analyses in chemical ecology are performed in Python or R, which provide extensive libraries for data preprocessing, model development, visualization, interpretation, and reproducible analysis. Python is widely used for deep learning, molecular modeling, and scalable ML through libraries such as scikit-learn, XGBoost, LightGBM, TensorFlow, PyTorch, SHapley Additive exPlanations (SHAP), and RDKit (Pedregosa et al. 2011, Chen and Guestrin 2016). R remains widely used for statistical modeling, multivariate analyses, and ecological data analysis through packages, including tidymodels, caret, randomForest, ranger, mlr3, mixOmics, and vegan (Kuhn 2008, Rohart et al. 2017, Lang et al. 2019). These open-source tools enable researchers to develop reproducible workflows for analyzing chemical, behavioral, electrophysiological, and environmental datasets without relying on proprietary software.

**Table 1.** Summary of the major machine learning paradigms, their primary analytical objectives, the types of data they analyse, and representative applications in chemical ecology and integrated pest management

ML paradigm	Typical data	Primary objective	Representative chemical ecology/IPM application
**Unsupervised learning**	Unlabeled volatile profiles, cuticular hydrocarbons, electrophysiological responses, behavioral trajectories	Pattern discovery, clustering, and dimensionality reduction	Identifying chemical phenotypes, blend structure, behavioral groups
**Supervised learning**	Labeled volatile, electrophysiological, behavioral, sensor, and field data	Classification, regression, and prediction	Predicting semiochemical activity, odorant receptor ligand identification, pest detection
**Generative learning**	Molecular structures, SMILES, MS/MS spectra, chemical descriptors	Molecule generation and structure elucidation	Designing novel semiochemicals, identifying unknown compounds
**Optimization-based learning**	Spatial, temporal, environmental, and monitoring data	Decision optimization	Optimizing trap placement, mating disruption, attract and kill deployment, intervention timing

The following sections describe how each ML paradigm addresses specific analytical challenges in chemical ecology and supports different stages of semiochemical discovery, optimization, deployment, and decision support.

#### Unsupervised Learning: Discovering Latent Structure in Unlabeled Data

Unsupervised learning identifies structure in data without predefined output labels (Hastie et al. 2009). These methods are exploratory, revealing hidden organization in complex datasets, supporting hypothesis generation, and reducing dimensionality for interpretation (Hervé et al. 2018). In chemical ecology, biologically relevant chemical signals are often unknown, and behavioral outcomes are only partially characterized. Unsupervised learning is therefore well suited to volatile profiles (Zung et al. 2023), cuticular hydrocarbons (CHCs) (Baleba et al. 2024), electrophysiological responses (Date et al. 2013, Crowley-Gall et al. 2025), and fluorescence spectra (Niewalda et al. 2011, Strauch and Galizia 2012). This section considers 6 methodological families. These include (i) dimensionality reduction methods such as principal component analysis (PCA), nonmetric multidimensional scaling (NMDS), principal coordinates analysis (PCoA), *t*-distributed stochastic neighbor embedding (*t*-SNE), and uniform manifold approximation and projection (UMAP), (ii) clustering algorithms, (iii) matrix factorization methods such as non-negative matrix factorization (NMF), (iv) neural network based approaches such as self-organizing maps (SOM), and (v) blind source separation methods such as independent component analysis (ICA), and (iv) latent variable regression methods such as Partial Least Squares (PLS). It is important to note that PLS is not strictly an unsupervised method. It performs dimensionality reduction by extracting latent chemical structure, but component estimation is explicitly constrained by biological response variables.

##### Dimensionality reduction techniques.

Dimensionality reduction methods produce low-dimensional representations of complex chemical or sensory datasets. They are primarily used to visualize sample structure and identify chemical dimensions that drive biological separation, generate hypotheses, and support feature selection before predictive modeling. All ordination approaches are sensitive to data scaling and preprocessing. Careful preprocessing, including peak deconvolution, retention index verification, compound annotation, and quality control, is therefore essential because systematic errors in chemical data propagate through downstream analyses and can compromise model performance and biological interpretation. PCA identifies orthogonal axes that explain decreasing proportions of total variance. Park et al. (2020) applied PCA to volatile profiles of sorghum plants infested by the sugarcane aphid *Melanaphis sacchari* (Zehntner) (Hemiptera: Aphididae). Although compound identities did not differ qualitatively, PCA separated healthy and infested plants based on quantitative differences in volatile composition. This guided subsequent classification and improved treatment discrimination. PCA can also link chemical variation directly to insect behavior. Pareja et al. (2009) reduced plant volatile profiles to principal components and used them to predict the attraction of the parasitoid *Aphidius ervi* (Haliday) (Hymenoptera: Braconidae) in *Acyrthosiphon pisum* (Harris) (Hemiptera: Aphididae) systems on alfalfa and broad bean. In both plant species, a single principal component explaining only a small proportion of the total variance was the strongest predictor of parasitoid attraction. This finding suggested that minor blend components, rather than dominant volatiles, can drive behavioral responses and provided a basis for prioritizing compounds for behavioral assays.

NMDS uses rank-based dissimilarities and accommodates any distance metric (Souza 2025). Like PCA, NMDS is an established ordination method that is widely used to explore multivariate chemical ecology datasets before applying predictive ML approaches. Its main limitations are dependence on the selected distance measure and instability when stress values are high. Baleba et al. (2024) used NMDS with Bray–Curtis dissimilarity to visualize CHC profiles of 3 *Drosophila* species (Diptera: Drosophilidae) reared at 15 to 30 °C. NMDS clearly separated samples by temperature for all species, demonstrating its utility for visualizing phenotypic plasticity along environmental gradients. Similarly, Crandall et al. (2025) applied NMDS to beetle community responses to fungal volatiles, host stress odors, and pheromones, where ordination revealed treatment separation supported by permutational multivariate analysis of variance and identified blend types that restructure community assembly. This directly informs candidate blend selection for field manipulation. Likewise, Peter et al. (2023) used NMDS to map volatiles across host and companion plants, identifying key discriminating compounds and linking chemical variation to *Spodoptera frugiperda* (Smith) (Lepidoptera: Noctuidae) behavior, including visitation, landing distance, and oviposition, which was associated with reduced infestation in a maize-based intercropping system. At the population scale, Ramírez et al. (2010) used NMDS to analyze fragrance profiles of male orchid bees (Hymenoptera: Apidae: Euglossini) and demonstrated differentiation between native and introduced populations. This provided evidence that environmental chemical availability shapes acquired scent profiles.

Principal Coordinate Analysis operates on distance matrices and enables inspection of compound or response loadings using biplots. Interpretation can be difficult when multiple dissimilarity measures are possible. Erdei et al. (2025) applied PCoA to antennal response profiles of *Drosophila melanogaster* (Meigen) and the variegated fruit fly, *Phortica variegata* (Fallén) (Diptera: Drosophilidae). The analysis separated species and identified compounds driving the separation. This guides ligand selection for comparative electrophysiology. Yoneya et al. (2012) found that predatory insects respond differently to willow volatiles depending on the herbivore feeding on the plant. PCoA ordination separated volatile blends from different willow–herbivore combinations. Variation along the main PCoA axes correlated with predator attraction, highlighting the chemical differences that drive behavior. This identifies behaviorally relevant dimensions of plant chemical variation and clarifies how plants indirectly recruit natural enemies, informing conservation biological control strategies.

*t*-SNE is a nonlinear method that preserves neighborhood structure. It is effective for detecting compact clusters but is sensitive to parameter settings and run-to-run variability. Caballero-Vidal et al. (2020) applied *t*-SNE to molecular descriptors of odorants tested against the *Spodoptera littoralis* receptor OR25. Agonists and nonagonists formed distinct clusters, guiding large-scale virtual screening. Yoshida and Toyoizumi (2025) showed that *t*-SNE applied to olfactory receptor response matrices produces functional group separation that supports hypotheses on odor coding by sensory circuits. UMAP preserves local and global structure and allows projection of new samples into an existing embedding. These properties are useful for longitudinal and multiexperiment chemical ecology datasets. Its main limitations are sensitivity to parameter settings and dependence on the selected distance metric. Nguyen et al. (2026) applied UMAP to calcium imaging data from mosquito antennal neurons responding to multiple odorants. UMAP separated response patterns across stimuli and revealed structure in representations of repellents and natural odors. This supports the use of UMAP to prioritize candidate volatiles for behavioral testing based on sensory-response structure.

Collectively, dimensionality reduction techniques transform complex chemical, electrophysiological, and behavioral datasets into interpretable representations that facilitate hypothesis generation and biological interpretation. By revealing the principal dimensions underlying chemical variation and sensory responses, these methods help prioritize candidate semiochemicals, identify behaviorally relevant patterns, and guide subsequent behavioral and field validation within integrated pest management programs.

##### Clustering algorithms.

Clustering methods assign samples to discrete groups based on similarity. They identify chemotypes, ecological states, and treatment-specific signatures. Cluster solutions depend on the choice of algorithm and parameter settings. Stability assessment is therefore essential. In this context, M’Sakni et al. (2025) applied hierarchical clustering to volatile fingerprints in pomegranate-based multitrophic systems involving *Aphis punicae* (Passerini) (Hemiptera: Aphidinae), *Tapinoma magnum* (Mayr) (Hymenoptera: Formicidae), and *Coccinella undecimpunctata* (L.) (Coleoptera: Coccinellidae). The analysis revealed scenario-specific clusters and stage-dependent volatile changes (M’Sakni et al. 2025). Suppression of β-farnesene and methyl salicylate was detected during early infestation, while the ant pheromone 4-heptanone appeared after 24 h, and an increase in herbivore-induced volatiles after 48 h (M’Sakni et al. 2025). These patterns directly support stage-specific blend design. Jamil et al. (2024) combined hierarchical clustering, k-means and DBSCAN to group sites based on stingless bee communities and environmental variables. The resulting clusters defined the environmental contexts for stratifying field sites before chemical ecology experiments (Jamil et al. 2024).

Overall, clustering algorithms reveal natural groupings within chemical ecology datasets without requiring predefined classes. They support the identification of chemotypes, ecological states, and environmentally driven response patterns, providing a rational basis for targeted semiochemical discovery, experimental design, and site stratification in IPM studies.

##### Self-organising maps.

The SOM projects high-dimensional data onto a discrete grid while preserving topological relationships, combining visualization with clustering (Agboka et al. 2025, 2026). A key limitation is the need to predefine grid size and training parameters. Steiner et al. (2002) applied SOM to CHC profiles of *Tetramorium* ants, revealing chemical phenotypes that aligned with species boundaries and uncovered multiple profiles within morphologically similar taxa. This generated hypotheses of cryptic species or hybridization and guided targeted taxonomic and behavioral validation. SOMs combine dimensionality reduction with clustering, enabling intuitive visualization of complex chemical datasets while preserving biologically meaningful relationships among samples. Their ability to uncover hidden chemical phenotypes makes them valuable for hypothesis generation, comparative analyses, and prioritizing taxa or populations for further behavioral and ecological investigation.

##### Non-negative matrix factorisation.

NMF decomposes signals into additive, non-negative components that are directly interpretable. Selection of the number of components remains critical. Harada and Nakamoto (2014) applied NMF to olfactory receptor neuron responses, extracting components that represented odor basis patterns and enabled reconstruction of complex fruit odor responses. This approach supports targeted reconstruction of blend components relevant for sensory coding and behavioral testing. By decomposing complex responses into additive and interpretable components, NMF facilitates the identification of underlying sensory features that contribute to odor perception. This provides a useful framework for reconstructing behaviorally relevant odor blends and simplifying complex chemical mixtures for downstream validation.

##### Independent component analysis.

ICA separates mixed signals into statistically independent components and is designed for blind source separation. Major limitations include component interpretation and sensitivity to noise. Cordella et al. (2019) applied ICA to 3-dimensional fluorescence spectra of honeybees exposed to dimethoate. Independent components corresponded to neurotransmitter-related signals and stress-associated fluorophores. This enabled rapid identification of physiological pathways affected by pesticide exposure. ICA is particularly valuable when chemical or physiological measurements arise from multiple overlapping biological processes. By separating independent signal sources, it improves the interpretation of complex datasets and enables identification of biologically meaningful components that may otherwise remain obscured.

##### PLS and related latent variable regression.

PLS extracts latent variables that maximize covariance between chemical predictors and biological responses (Panjwani et al. 2025). It combines dimensionality reduction and prediction and is therefore often perceived as intermediate in use, although it remains a supervised method. PLS does not identify latent structure independently of biological responses. It extracts a chemical structure constrained by biological performance. However, overfitting remains a limitation when sample sizes are small. For example, D’Acqui (2017) used PLS to predict foliage chemistry and performance indices of *Lymantria dispar* (L.) (Lepidoptera: Erebidae) and *Malacosoma neustrium* (L.) (Lepidoptera: Lasiocampidae). The analysis linked spectral measurements to insect physiological performance and enabled rapid screening of host plant quality. In addition, Jeria et al. (2017) applied unfolded PLS with residual bilinearization to quantify imidacloprid in honeybees despite strong matrix interference. This demonstrates how latent regression can recover chemically meaningful signals from complex biological matrices. Unlike purely exploratory dimensionality reduction methods, PLS directly links chemical predictors with biological responses, making it particularly useful when the objective is prediction rather than pattern discovery. This capability supports rapid screening of candidate semiochemicals and facilitates the translation of complex chemical measurements into biologically relevant and operational IPM outcomes.

#### Supervised Learning: From Labeled Data to Predictive Models

Traditional workflows for identifying behaviorally active semiochemicals are time-consuming and resource-intensive (Barbosa-Cornelio et al. 2019). They rely on repeated cycles of chemical separation, electrophysiology, and behavioral bioassays. van Dam and Poppy (2008) proposed using ML in chemical ecological research to address these constraints. They noted that insects detect specific volatiles within complex mixtures, a process conceptually analogous to feature selection and dimension reduction in supervised algorithms, such as SVM and RF. Supervised learning includes algorithms that learn relationships between input features and known output labels. Once trained, these models predict outcomes for untested samples. They are therefore suited to classifying semiochemical activity, modeling structure–activity relationships, and decoding electrophysiological or behavioral signals. In practice, supervised learning supports experimental prioritization, accelerates compound discovery, and automates signal interpretation (Pathak et al. 2025).

##### Predicting semiochemical activity from chemical structure.

A central application of supervised learning is the prediction of behavioral or physiological activity of untested volatile compounds from molecular structure. In the tobacco cutworm, *S. littoralis* (Boisduval) (Lepidoptera: Noctuidae), Caballero-Vidal et al. (2020) trained an SVM using structural descriptors of known odorant receptor (OR) ligands to predict new agonists. Experimental testing of 32 predicted compounds identified 11 novel receptor agonists, representing a success rate of 28%, thereby demonstrating that ML can efficiently prioritize biologically active semiochemicals for functional validation while greatly reducing experimental screening effort. However, these compounds were validated only in receptor-based laboratory assays and were not evaluated under field conditions. Similarly, Tauxe et al. (2013) used SVM to screen more than 440,000 compounds for mosquito attractant and repellent activity. One predicted attractant, cyclopentanone, was subsequently validated in semi-field trapping experiments with *Culex quinquefasciatus*, where it attracted mosquitoes at levels comparable to carbon dioxide despite being released at substantially lower quantities. This study demonstrates that ML predictions can translate into operationally relevant attractants beyond laboratory assays. Santos and Janairo (2025) developed ML classification models using molecular complexity and structural descriptors to predict electroantennogram activity in the red-necked longhorn beetle. Their models identified nonlinear relationships between molecular complexity and semiochemical activity and used SHAP analysis to quantify the contribution of individual molecular descriptors, providing mechanistic insights that can guide future semiochemical discovery and experimental validation. The study did not include behavioral or field validation of newly predicted compounds. Beyond classification of known compounds, AI-driven approaches such as deep learning encoder–decoder models can perform de novo structural elucidation directly from tandem mass spectrometry spectra, enabling the identification of truly novel semiochemicals that fall outside existing chemical databases and would otherwise remain structurally uncharacterized (Stravs et al. 2022).

##### Decoding electrophysiological and behavioral responses.

Supervised learning has also been applied to high-dimensional electrophysiological data to automate the interpretation of neural responses. For example, Swore et al. (2025) trained SVM and RF classifiers on electroantennogram recordings from *M. sexta* to discriminate responses to single odorants and mixtures. This increased analytical throughput and reduced subjective interpretation. For behavioral data, Fazzari et al. (2024) combined pose estimation with deep learning to classify antennal movement patterns in the house cricket *Acheta domesticus* (L.) (Orthoptera: Gryllidae) exposed to different chemical stimuli. The resultant models distinguished responses to sucrose and ammonia from movement sequences alone, thus providing a scalable framework for automated decoding of chemically induced behavior. Despite advancements in pose estimation and classification methods, significant limitations hinder their broader applicability. The generalization of models across diverse species and conditions is lacking, as species-specific anatomical configurations limit transferability. Additionally, supervised learning approaches are constrained by the limited availability of training data in chemical ecology, leading to overfitting and reduced model robustness. The physiological constraints of the EAG signal, which reflects collective neuron activity, obscure the detailed neural coding necessary for olfactory discrimination. Furthermore, both SVM and deep learning classifiers do not provide insights into the specific chemical features or pathways involved, limiting mechanistic understanding in chemical ecology.

##### Predicting ligands for olfactory receptors and binding proteins.

Supervised learning has been used to predict which volatiles activate specific olfactory receptors or bind to odorant binding proteins. Caballero-Vidal et al. (2021) trained classifiers to identify receptor agonists in *S. littoralis*. Predicted compounds were confirmed in physiological and behavioral assays. At the protein level, López Cortés et al. (2025) applied gradient boosting and XGBoost to predict binding affinities between *M. sexta* pheromone-binding proteins and volatile compounds. Their XGBoostRegressor achieved an *R*² of 0.76, enabling targeted selection of ligands for costly experimental validation.

##### Discovering antagonists of conserved olfactory targets.

Supervised learning has also enabled the discovery of antagonists targeting the conserved olfactory co-receptor Orco. Kepchia et al. (2019) trained models using primary screening data to rank candidate Orco inhibitors in *Anopheles gambiae* Giles (Diptera: Culicidae). Highly ranked compounds inhibited receptor function in electrophysiological assays and altered behavior. This workflow focuses experimental effort on computationally prioritized candidates and accelerates repellent discovery.

RF: A Widely Adopted Tool for Volatile Analysis: RF is a tree-based ensemble method widely used in chemical ecology because it handles mixed feature types, nonlinear relationships, and correlated predictors while providing measures of variable importance (Ranganathan and Borges 2010). The latter authors showed that RF is well suited for volatile analysis because it (i) handles more volatiles than samples, (ii) maintains classification performance in the presence of noise, (iii) identifies minimal predictive subsets of volatiles, (iv) remains robust to interactions and correlations among predictors, and (v) ranks volatiles by relative importance.

In *Glossina* spp., RF distinguished volatiles from preferred and nonpreferred hosts and highlighted ketones and other VOCs as key host-seeking cues (Orubuloye et al. 2025). In the koinobiont parasitoid, *Dolichogenidea gelechiidivoris* (Marsh) (Hymenoptera: Bracionidae), RF ranked VOCs from healthy and *Phthorimaea* (=*Tuta*) *absoluta* (Meyrick)-infested tomato plants and identified α-pinene, β-myrcene, α-phellandrene, α-terpinene, β-ocimene, methyl salicylate, and (E)-β-caryophyllene as candidate attractants, later validated in Y-tube assays (Ayelo et al. 2022). In the tritrophic system involving tomato, *T. absoluta*, and the zoophytophagous predator *Nesidiocoris tenuis* (Reuter) (Hemiptera: Miridae), RF identified monoterpenes associated with herbivore and predator attraction and sesquiterpenes associated with nonhost repellence in *T. absoluta* (Adams et al. 2023). In *D. melanogaster*, RF classified fecal volatiles to distinguish carnivore from herbivore feces and identified phenol and indole as predictors of oviposition avoidance (Mansourian et al. 2016). In wild tomato avoidance, RF combined with sparse partial least squares discriminant analysis (sPLS-DA) and SIMPER identified repellent allelochemicals, including (Z)-3-hexenol, camphor, citronellal, and limonene oxide, which were confirmed in GC EAG and wind tunnel assays (Miano et al. 2022). Peterson et al. (2020) applied RF to host selection in *Agrilus planipennis* (Fairmaire) (Coleoptera: Buprestidae). From 59 detected compounds across 5 host plants, RF identified 8 volatiles that best discriminated against plant species with an error rate of approximately 19%. Baleba et al. (2019) used RF to identify volatiles predicting oviposition substrate choice in the stable fly, *Stomoxys calcitrans* (L.) (Diptera: Muscidae). RF selected β-citronellene and carvone as predictors of donkey and sheep dung. Behavioral assays confirmed increased oviposition and higher trap captures. McCormick et al. (2016) used RF to identify volatiles used by *L. dispar* larvae to discriminate damaged from undamaged *Populus nigra* (L.) (Salicaceae) leaves. De Moraes et al. (2014) applied RF to identify volatiles used by the Asian malaria mosquito *Anopheles stephensi* (Liston) (Diptera: Culicidae) to discriminate *Plasmodium chabaudi*-infected from healthy mice.

##### Odorant receptor discovery.

Insect OR gene discovery has historically been labor intensive, relying on manual curation of antennal transcriptomes and genome annotations. This process typically misses 30% to 40% of gene content due to high evolutionary divergence, taxon specificity, and dynamic birth and death patterns within this family (Karpe et al. 2021). Modern ML approaches now accelerate this pipeline. Specialized tools such as InsectOR apply sensitivity-optimized homology-based algorithms to newly sequenced genomes and transcriptomes. With large-scale initiatives such as the Earth BioGenome Project generating thousands of new insect genomes, automated OR discovery is projected to reduce manual curation effort by approximately 75%. Beyond gene identification, ML has also been applied to functional annotation. Mier et al. (2022) analyzed 3,902 OR sequences from 21 species, using ML on functionally characterized proteins from *D. melanogaster*, *A. gambiae*, and *Harpegnathos saltator* (Jerdon) (Hymenoptera: Formicidae) to identify amino acid positions that best predict ligand response. This provides a route to infer OR function from sequence alone, bypassing extensive laboratory screening. Importantly, linking receptor sequences to ligand specificity accelerates the identification of behaviorally active semiochemicals, facilitating the development of novel attractants, repellents, and monitoring lures for pest and vector management. Together, these approaches transform OR discovery from a specialized, manual bottleneck into a scalable, computationally driven process, enabling rapid translation of genomic resources into practical chemical ecology applications and odor-based integrated pest management strategies.

##### Matching algorithms to data characteristics.

The choice of a supervised algorithm should follow the dataset characteristics. SVM performs well with smaller datasets and well-separated classes when feature engineering is appropriate (Cervante et al. 2020). Tree-based ensembles such as RF and gradient boosting handle nonlinear relationships, mixed predictors, and correlated features, and are well-suited to tabular chemical datasets (Breiman 2001, Friedman 2001, Taha 2025). Deep neural networks can learn representations directly from molecular structures or time series signals but typically require larger datasets than are available in most exploratory chemical ecology studies (Borowiec et al. 2022, Kumar and Srivastava 2024). In practice, systematic comparison of multiple supervised algorithms using cross-validation and independent test sets provides a robust framework for model evaluation and selection (Arlot and Celisse 2010, Wilimitis and Walsh 2023). In addition, combining multiple models through ensemble approaches can further improve predictive stability and reduce variance, particularly in high-dimensional and noisy datasets (Amr et al. 2026, Saha et al. 2026). However, gains in performance should be balanced against interpretability, and all approaches should be validated using independent test sets.

Across applications, including semiochemical activity prediction, electrophysiological signal classification, behavioral decoding, ligand discovery, and antagonist identification, supervised learning transforms labeled observations into predictive models that directly support experimental prioritization and signal interpretation. These approaches strengthen the efficiency and predictive capacity of chemical ecology research. These unsupervised and semi-supervised approaches are complementary. Ordination methods generate hypotheses on chemical dimensions and treatments that structure biological responses. Clustering identifies discrete chemical and ecological states. SOM reveals topological organization and hidden phenotypes. NMF and ICA decompose complex sensory and physiological signals into interpretable components. PLS links chemical variation to biological performance. Together, these methods enable the detection of herbivore-induced changes not detected by univariate analyses, identification of low variance blend components that drive behavior, visualization of community level responses to volatile manipulation, linking of plant chemical variation to predator and parasitoid attraction, identification of cryptic chemical phenotypes, and isolation of neurochemical stress responses. When combined with behavioral and electrophysiological validation, unsupervised learning provides a practical engine for hypothesis generation and compound prioritization in chemical ecology.

### Predictive and Generative Modeling for Semiochemical Discovery

**Fig. 4.**
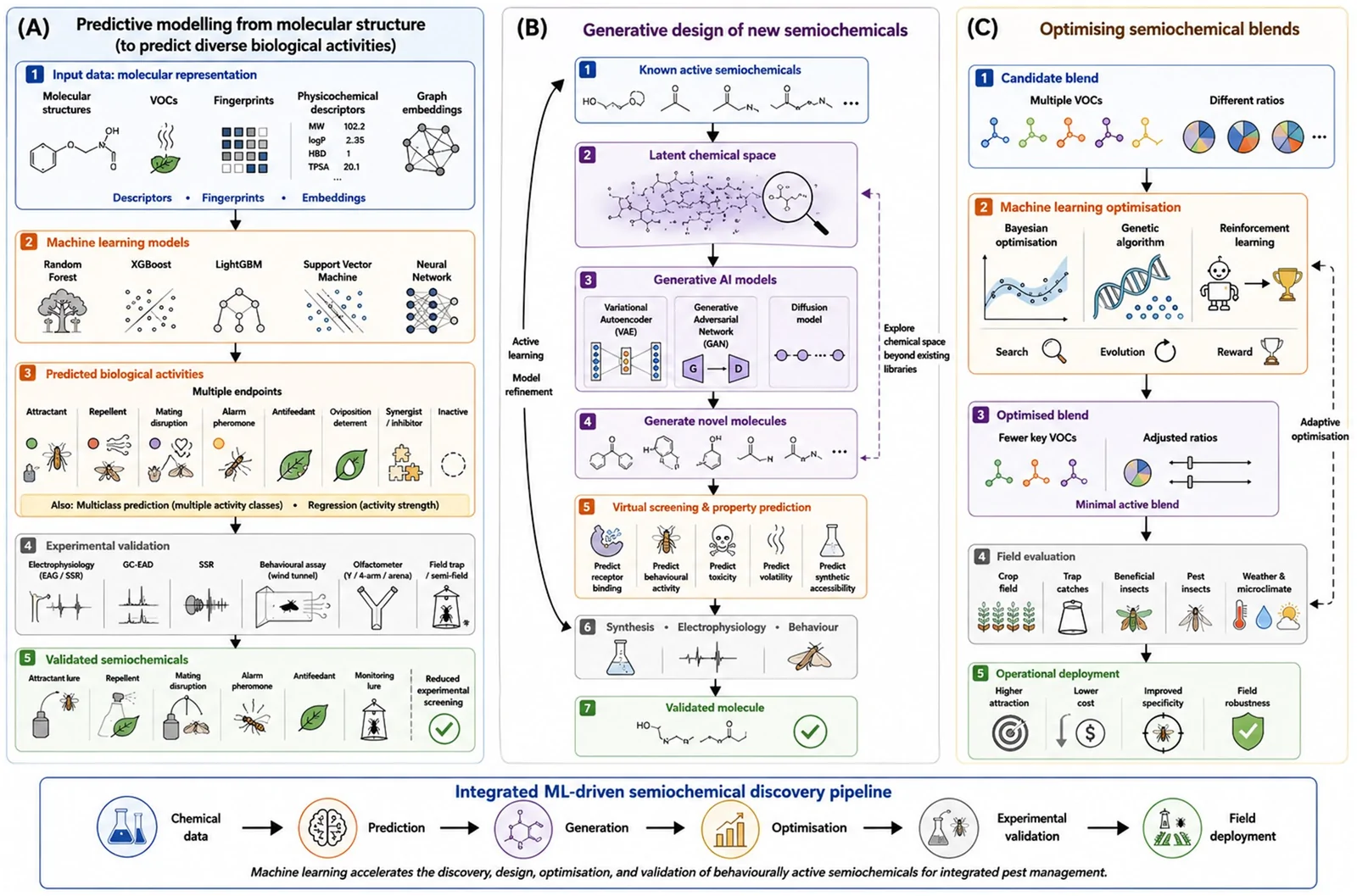
Machine learning approaches for semiochemical discovery and optimization. A) Predictive models identify biologically active semiochemicals from molecular representations and prioritize candidates for experimental validation. B) Generative AI designs novel semiochemicals through exploration of latent chemical space followed by virtual screening and iterative model refinement. C) Optimization algorithms identify effective semiochemical blends and refine deployment through adaptive field validation, accelerating the development of semiochemical based integrated pest management strategies.

Discovery of behaviorally active semiochemicals traditionally relies on labor-intensive workflows, including bioassay-guided fractionation, electrophysiological screening, and field validation (Bruce et al. 2005, Pickett et al. 2012, 2016, Jayanthi et al. 2014). These approaches have yielded many attractants and repellents but are difficult to scale across the vast chemical diversity of VOCs involved in insect olfaction (Carey and Carlson 2011, Riffell 2012, Riffell et al. 2014, Conchou et al. 2019). ML provides an efficient alternative. Algorithms can predict behavioral activity from molecular structure (Fig. 4A), generate novel candidates (Fig. 4B), and optimize semiochemical blends before empirical testing (Fig. 4C) (Noé et al. 2020, Alencar Filho et al. 2025). This reduces the search space and accelerates the identification of ecologically relevant compounds (Kepchia et al. 2019, Caballero-Vidal et al. 2021).

Early studies attempted to link molecular structure to olfactory perception using small odorant datasets and limited descriptors, with modest predictive power (Amoore 1963, Dravnieks 1972, Schiffman 1974, Haddad et al. 2008). Contemporary ML uses thousands of descriptors and large chemical libraries, often with neural networks that learn representations from molecular graphs (Rogers and Hahn 2010). A key challenge remains the absence of a defined odor metric space. Models should link molecular structure to neural responses and behavioral outcomes shaped by ecological interactions (Haddad et al. 2008). ML addresses this challenge by embedding molecules in high-dimensional physicochemical spaces where distances correspond to predicted activity differences (Fig. 4A). This approach, using ML to learn predictive relationships between molecular structure and olfactory activity from large datasets, has recently been extended into integrated discovery pipelines (Fig. 4, stages 1 to 5) that combine predicted odorant structures, large-scale docking, and ML classification to identify behaviorally active ligands from vast candidate sets (Jin et al. 2023, Comte et al. 2025). This reverse chemical ecology approach uses receptor targets to explore chemical space without assumptions about candidate sources. Molecular representations—descriptors, fingerprints, and learned embeddings, define computational spaces where similarity guides predictions (Fig. 4A) (Rogers and Hahn 2010). Effective representations capture receptor binding, volatility, and ecological relevance, underpinning both predictive and generative strategies (Fig. 4A and B).

### Regression and Classification for Behavioral Activity Prediction

Predicting behavioral responses from molecular structure is central to semiochemical discovery (Fig. 4A). Quantitative structure–activity relationship (QSAR) modeling frames this as a statistical association between molecular features and biological outcomes (Gramatica 2007, Tropsha 2010, Cherkasov et al. 2014). This approach reduces large chemical spaces into smaller, prioritized candidate libraries for experimental validation. Supervised learning requires curated datasets linking molecular structures to behavioral or molecular interaction outcomes. Endpoints can be binary (attractant vs nonattractant), multiclass (eg attraction, repellency), or continuous (receptor activation magnitude). Features include physicochemical descriptors (molecular weight, logP), structural fingerprints (Morgan, MACCS), and learned graph embeddings (Fig. 4A) (Rogers and Hahn 2010, Baptista et al. 2022).

High-dimensional descriptor spaces capture diverse structural attributes but risk overfitting when sample sizes are limited. Dimensionality reduction and feature selection are essential (Sheridan 2013, Vamathevan et al. 2019). Tree-based ensembles such as Random Forest, XGBoost, LightGBM, and CatBoost capture nonlinear relationships and interactions while providing feature importance metrics (Breiman 2001, Chen and Guestrin 2016). For example, XGBoost predicted attractant volatiles in *Ceratitis capitata* (Wiedemann) (Diptera: Tephritidae), highlighting features associated with behavioral responses (Alencar Filho et al. 2025). Fundamentally, SVMs remain effective for smaller datasets with clear class separation (Cortes and Vapnik 1995). In moth olfaction, SVMs classified *M. sexta* electroantennogram responses, thereby automating the discrimination of signals correlated with compound identity and concentration (Swore et al. 2025).

Electrophysiological data expands model utility. EAG, GC-EAD, and SSRs capture neural response patterns that lie intermediate between molecular structure and behavior (Caballero-Vidal et al. 2021, Comte et al. 2025). Large-scale virtual screening demonstrates the reach of these approaches (Fig. 4, stages 3 and 4). SVMs trained on mosquito repellents can identify structurally diverse carbon dioxide receptor antagonists and be behaviorally validated as described in Tauxe et al. (2013) and Kowalewski et al. (2024). Predictive models trained on the OR25 ligands of *S. littoralis* prioritized novel agonists from chemical libraries, confirmed through electrophysiology and behavior (Caballero-Vidal et al. 2021).

Docking workflows require careful scoring. Standard scoring can favor large molecules, while rescoring by ligand efficiency improves true binder discrimination (Jin et al. 2023, Comte et al. 2025). AlphaFold2-predicted receptor structures improve docking even with low template identity. Small, fragmented datasets remain a limitation, increasing overfitting and reducing generalization (Barwich and Lloyd 2022). Standardization, integration of multimodal observations, and open-access datasets are critical. Explainable AI methods, including feature importance and SHAP values, enhance interpretability and guide targeted synthesis (Murdoch et al. 2019).

### Generative Models for Novel Semiochemical Design

Predictive models can prioritize known molecules but remain limited in existing libraries. Generative modeling overcomes this limitation by learning the distribution of active compounds and propositions of new molecular structures (Fig. 4B) (Olivecrona et al. 2017, Bilodeau et al. 2022). This shift enables de novo design of semiochemicals tailored to biological targets and operational constraints. Generative frameworks include variational autoencoders (VAEs), generative adversarial networks (GANs), and reinforcement learning strategies (Fig. 4B). VAEs encode molecular structures into continuous latent spaces, enabling sampling and reconstruction of novel molecules (Ochiai et al. 2023). This representation supports navigation between known actives and the generation of intermediates. GANs use a generator and a discriminator to produce realistic structures (Tripathi et al. 2022). These models enable exploration of chemical space beyond natural or synthetic libraries. Unlike virtual screening, which evaluates existing molecules, generative models propose compounds that have not been synthesized (Stanley and Segler 2023). They generate structures linked to activity while remaining distinct from known compounds. In semiochemicals discovery, this supports the design of volatiles with defined receptor activation and volatility (Bilodeau et al. 2022). Models trained on known attractants can generate analogues with improved stability or reduced cost while preserving activity (Elton et al. 2019). Generative approaches can include constraints, such as environmental stability, toxicity, and synthetic accessibility (Olivecrona et al. 2017, Polykovskiy et al. 2020, Alam et al. 2025). Multiobjective frameworks can optimize receptor affinity, nontarget safety, environmental persistence, and synthetic routes before synthesis (Gómez-Bombarelli et al. 2018, Jensen 2019).

The broader field of generative chemistry provides methods transferable to semiochemical discovery. VAEs, GANs, and transformer models generate chemically valid molecules that respect valency constraints (Gómez-Bombarelli et al. 2018, Zhavoronkov et al. 2019). These models learn chemical rules from data and produce synthesizable candidates in new regions of chemical space. Retraining datasets of behaviorally active volatiles enables targeting insect olfactory systems. Sharma et al. (2025) employed graph generative models to navigate fragrance-relevant chemical space, generating novel molecular structures while simultaneously predicting their odor likeliness with high accuracy (ROC Area Under the ROC Curve [AUC] = 0.97) and assigning probable odor labels. The generated molecules exhibited physicochemical properties consistent with known odorants, and the authors leveraged SHAP to identify chemical features most strongly associated with olfactory activity. Related approaches use conditional VAEs to generate molecules tailored to receptor binding sites with predicted affinity (Gómez-Bombarelli et al. 2018). Extending these frameworks to insect ORs is now feasible.

Validation pipelines in insect systems are established. Caballero-Vidal et al. (2020, 2021) showed that ML can screen millions of compounds and identify new agonists for the *S. littoralis* receptor SlitOR25. Of the 3 million candidates, 32 were tested and 9 to 11 validated electrophysiologically. Kepchia et al. (2019) identified behaviorally active antagonists of the Orco receptor using virtual screening, electrophysiology, and behavioral assays. López-Cortés et al. (2025, 2026) predicted interactions between volatiles and odorant binding proteins using combined protein and ligand features. Progress in olfaction modeling further supports generative design. Continuous odor spaces can be learned from molecular data (Schmuker and Schneider 2007, Haddad et al. 2008). These representations guide molecule generation. Graph-based models can predict properties of odor mixtures and capture nonlinear interactions (Schmuker and Schneider 2007), enabling the design of both compounds and blends.

However, no study has yet demonstrated the full chain from generative design to receptor activation and behavioral validation in insects. Key components include predictive screening models (Caballero-Vidal et al. 2020, 2021), experimental validation pipelines (Kepchia et al. 2019, Comte et al. 2025), generative frameworks (Gómez-Bombarelli et al. 2018), and structured odor spaces (Haddad et al. 2008). Integrating these elements defines a clear next step (Fig. 4, stages 1 to 5). Conditional models can generate ligands specific to single receptor tuning profiles. These frameworks can extend to blend design by targeting mixture-level interactions. Generation can be constrained by volatility ranges relevant to field conditions. Protein-informed approaches can guide design using receptor or binding protein structures (Comte et al. 2025). Generative models extend predictive pipelines. A typical cycle involves generation, prediction of activity and feasibility, experimental validation, and model refinement. This iterative process expands chemical space.

Current limitations include limited behavioral datasets, uncertainty in synthesis, and a lack of standardized validation pipelines. Active learning selects informative compounds and improves models. As datasets linking molecular structure to behavior expand, generative approaches will support targeted semiochemical design for pest management (Kepchia et al. 2019, Caballero-Vidal et al. 2020, 2021).

### Optimization of Blends for Biological and Operational Relevance

Insect behaviors often depend on volatile compound blends (Fig. 4C). These blends are not restricted to plant-emitted volatiles. Operational semiochemical systems frequently combine pheromones with kairomones or food-derived odors to increase attraction (Reddy and Guerrero 2004, Webster and Cardé 2017). For example, aggregation pheromones are commonly combined with food-derived attractants in monitoring programs for several stored product beetles, including *Rhyzopertha dominica* (Edde 2012), while predators and parasitoids often respond synergistically to herbivore induced plant volatiles together with herbivore-derived cues (Vet and Dicke 1992, Bruce et al. 2005). These examples illustrate the diversity of chemical blends that ML approaches must analyze and optimize for effective pest management. Insects often respond differently to complete volatile blends than to individual components, and the specific combinations and ratios determine host recognition and behavioral responses (Bruce and Pickett 2011). Plant volatile plumes vary in composition and ratio during release and transmission, affecting how insects track and interpret these signals to locate hosts or mates (Beyaert and Hilker 2014). These effects create a complex optimization problem across both compound identity and relative proportions. ML methods, such as Bayesian optimization, genetic algorithms, and reinforcement learning, are refining blend composition based on experimental outcomes (Shahriari et al. 2016, Olson and Moore 2019). Bayesian optimization builds surrogate models of response surfaces and efficiently identifies promising mixtures. Several studies illustrate these approaches in insect systems. Ayelo et al. (2022) used RF to identify volatiles mediating attraction of the parasitoid *D. gelechiidivoris* to *T. absoluta*-infested tomato plants, highlighting key compounds including α-pinene, β-myrcene, methyl salicylate, and (E)-β-caryophyllene, which were validated behaviorally. Adams et al. (2023) applied a similar approach to a tritrophic system involving tomato, *T. absoluta*, and *N. tenuis*, identifying compounds that attract natural enemies while repelling pests. Baleba et al. (2019) predicted oviposition in *S. calcitrans*, spotlighting β-citronellene and carvone, which increased trap captures. Peterson et al. (2020) reduced 59 host plant volatiles of *A. planipennis* to 8 discriminants, creating a tractable set for behavioral testing. Miano et al. (2022) applied unsupervised and supervised methods to reveal blends mediating avoidance behavior in female *T. absoluta*, highlighting the utility of multivariate statistical learning combined with electroantennogram data. Optimizing component ratios requires additional strategies. Bayesian optimization efficiently explores composition space and converges in a few iterations for small blends (Hickman et al. 2022, Shen et al. 2025). Reinforcement learning treats each blend as an action and the observed behavioral response as a reward, adapting compositions to maximize target outcomes. This approach has been applied as a sequential design strategy in experimental settings (Sridharan et al. 2024, Shen et al. 2025).

Structure-based insights further guide blend design. Structural features of insect ORs, including binding pocket geometry, residue composition, and flexibility, influence which ligands a receptor recognizes and how broadly it is tuned (Bohbot and Pitts 2015, Saberi and Seyed-allaei 2016, Li et al. 2026). Reviews on insect OR structure and function highlight how these determinants shape ligand selectivity and response properties (Ha and Smith 2022), providing a mechanistic context for compound selection. Comte et al. (2025) showed that broadly tuned receptors, for example, SlitOR25, bind diverse ligands through flexible hydrophobic contacts, whereas narrowly tuned receptors, including SlitOR31, use constrained hydrophobic and polar interactions. Across multiple receptors, binding pocket size and hydrophobicity correlated with tuning breadth (Pearson’s *r *= 0.4 to 0.6), and a simple model predicted receptor breadth with *r *= 0.75. These features inform the selection of compounds for specific receptor targets.

Moreover, ecological factors can also shape blend performance (Fig. 4, stages 4 and 5). Driving factors, such as temperature, humidity, background odors, and release kinetics, influence both signal emission and insect perception (Riveron et al. 2009, Webster and Cardé 2017, Baleba et al. 2023). Background odors can mask synthetic attractants, whereas blends differing from dominant crop volatiles can improve detection and trapping effectiveness (Reddy and Guerrero 2004, Beyaert and Hilker 2014). Laboratory optimization should link to field validation, as blends effective in controlled assays may fail under complex environmental conditions (Bruce et al. 2005, El-Sayed et al. 2009). Iterative workflows that incorporate field data improve robustness and accelerate optimization, enabling reliable operational formulations (Shahriari et al. 2016, Olson and Moore 2019). ML enables systematic blend optimization. Variable importance supports component selection, while optimization algorithms refine ratios, producing simpler, cost-effective blends that retain activity in the field.

### Integrated Deployment, Timing, and Modeling Strategies for Semiochemical-Based Pest Management

**Fig. 5.**
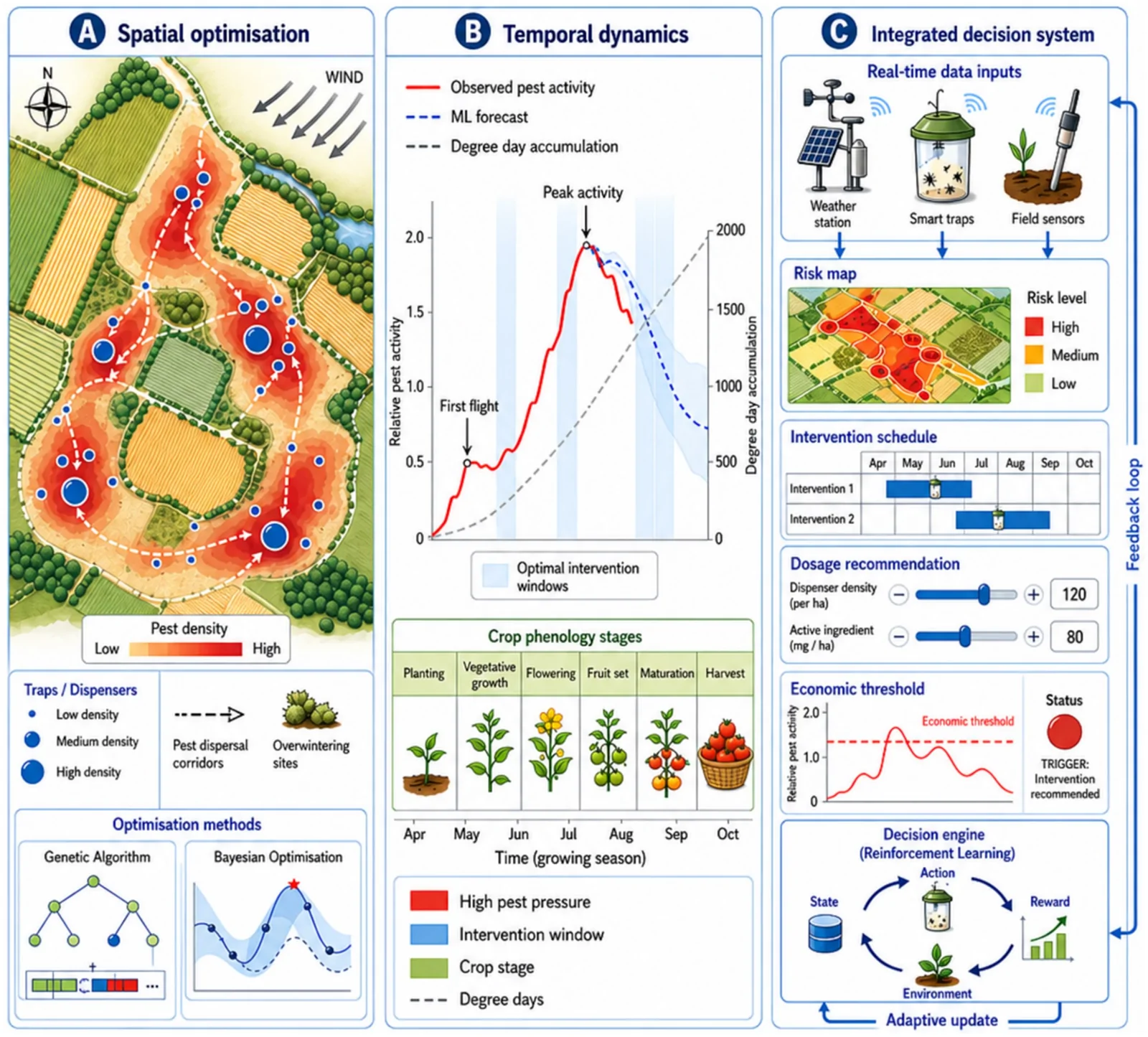
Spatial and temporal optimization for semiochemical deployment in pest management. A) Spatial optimization across heterogeneous landscapes. Panel shows crop fields, field margins, vegetation patches, and overwintering sites overlaid with a pest density heatmap. Trap and dispenser locations appear as blue circles scaled by deployment density. Arrows indicate wind direction, and curved paths show pest dispersal corridors. Optimization approaches such as genetic algorithms and Bayesian optimization allocate devices to high-risk zones and movement pathways while balancing coverage and resource constraints. B) Temporal dynamics and intervention timing. Panel presents pest population trajectories as a red curve across the growing season. Dashed gray line represents degree-day accumulation. Green bands mark crop phenology stages. Light blue vertical bands indicate optimal intervention windows derived from combined phenology models and machine learning forecasts. Predicted activity curves illustrate how integrating trap data and environmental variables improves the timing of deployment. C) Integrated decision system for adaptive management. Panel shows a dashboard combining spatial risk maps, intervention calendars, and dosage recommendations linked to economic thresholds. Real-time inputs from traps and environmental sensors feed predictive models that update recommendations through feedback loops. Reinforcement learning supports iterative decisions on where, when, and how much to deploy, enabling precise and adaptive pest management across space and time.

Deploying semiochemical interventions requires coordinated decisions on where, when, and how much to deploy (Witzgall et al. 2010, Baleba 2026). Spatial heterogeneity, temporal variation in pest dynamics, and environmental influences on semiochemical performance create a multidimensional optimization problem. Integrating spatial deployment, timing, dosage, and predictive modeling is essential for effective pest management (Fig. 5).

#### Spatial Deployment Across Heterogeneous Landscapes

Semiochemical tools such as mating disruption, mass trapping, and attract-and-kill systems must target areas where pest pressure varies with field margins, vegetation, overwintering sites, and dispersal corridors (Schumaker 1996, Ricci et al. 2009, Karp et al. 2018). These approaches rely on diverse classes of semiochemicals, including sex pheromones, aggregation pheromones, host plant volatiles, food kairomones, or combinations of these cues, depending on the target species and management objective. Effective placement balances coverage of high-risk areas, cost, and pest movement shaped by landscape structure (Holden et al. 2012). Spatial optimization methods provide systematic solutions: integer programming identifies optimal trap or dispenser locations under resource constraints; genetic algorithms explore continuous landscapes to maximize capture or coverage; Bayesian optimization refines configurations using observed trap data and spatial autocorrelation (Fig. 5A). Optimized pheromone networks for *Helicoverpa zea* (Boddie) (Lepidoptera: Noctuidae) reduced trap numbers while maintaining detection efficacy (Yanchenko et al. 2024). Similar models for *C. capitata* and *Anoplophora glabripennis* demonstrate how movement and landscape structure improve deployment efficiency (Manoukis et al. 2014). Push–pull strategies add spatial complexity, requiring placement of attractants and repellents relative to crops, trap crops, and natural enemies (Khan et al. 2011, Pickett et al. 2014, Sileshi et al. 2025). Environmental factors, including wind and dispenser density, further influence pheromone plume dispersion (Murlis et al. 2000).

#### Timing and Dosage Optimization

Deployment effectiveness depends on alignment with pest phenology and calibration to environmental conditions. Early, late, or insufficient application reduces impact, while excessive dosage increases costs or saturates sensory perception. Semiochemical release rates vary with temperature, humidity, and dispenser type (Zhu et al. 2015). Degree-day models estimate development from accumulated temperature and support first flight or peak activity predictions (Pruess 1983, Ebbenga et al. 2022), but linear assumptions limit accuracy under variable climates or complex life cycles (Barker and Coop 2023). Integrating phenology models with trap data improves activity window predictions (Rincon et al. 2024). ML captures nonlinear relationships; frameworks combining convolutional neural networks and LSTM models forecast pest pressure and identify optimal intervention windows (Kim and Sim 2025) (Fig. 5B). Hybrid ARIMA–LSTM models further improve temporal predictions (Wang and Li 2025). Field studies confirm the importance of timing: vineyard mealybug activity aligns with temperature ranges, showing that deployment within these windows enhances mating disruption efficacy (Daane et al. 2021). Dosage must balance efficacy, persistence, and cost, considering compound volatilization over deployment duration (Zhu et al. 2015).

#### Modeling Pest Dynamics and Crop Phenology

Accurate deployment relies on predicting pest populations and crop development. Mechanistic models, such as Leslie matrices for age-structured populations (Leslie 1945) or individual-based movement models (Bolliger 2007), guide landscape-level planning but require detailed parameterization. Data-driven models complement these by learning patterns from monitoring and environmental data. Integrated AI–Internet of Things trap systems enable real-time, proactive management (Passias et al. 2023). Combining remote sensing and meteorological data improves outbreak prediction (Chacón-Maldonado et al. 2025). Ensemble methods like RF and gradient boosting handle high-dimensional environmental data, improving accuracy across spatial scales (Singh et al. 2024, Mpisane et al. 2025). Explainable boosting models allow interpretation of key drivers while predicting rice pests such as the green leafhopper and the yellow stem borer (Wadhwa and Malik 2024). Hybrid approaches calibrate mechanistic parameters with ML, enhancing predictive accuracy and interpretability (Schmuker and Schneider 2007). Crop phenology affects susceptibility and volatile emissions, influencing semiochemical efficacy (Engelberth and Engelberth 2025, Thompson et al. 2026). Herbivore-induced volatiles vary with plant stage and damage intensity, and temporal shifts in chemical defenses highlight the need for seasonal adjustment (Mathur et al. 2011, Sanches et al. 2025). Remote sensing and environmental data enable prediction of phenology, aligning pest emergence with host susceptibility and defining optimal intervention windows.

#### Integration into Decision Systems

The full value of these approaches emerges when embedded in operational decision systems that translate predictions into action (Fig. 5C). Spatial optimization, phenology models, pest forecasts, and environmental data allow precise targeting across space and time. Explicit decision rules link forecasts to action thresholds based on pest density, trap captures, or predicted growth, informed by the Economic Injury Level framework (Pedigo et al. 1986, Hutchins et al. 1988). Probabilistic forecasts incorporate uncertainty, adjusting intervention intensity according to risk (Beddington et al. 1975, van den Bosch and Gilligan 2008). Continuous feedback and adaptive control, using automated traps and environmental sensors, enable iterative adjustment of strategies (Lewis et al. 1997, Damos 2015, Liu et al. 2016, Ferreira Lima et al. 2020, Ahmed et al. 2024). Reinforcement learning optimizes decisions over time, recommending where, when, and at what density to deploy while balancing immediate efficacy and long-term sustainability. Human-in-the-loop frameworks enhance usability, trust, and adoption (Rose et al. 2016, Lindblom et al. 2017). Spatial outputs such as maps indicate priority intervention zones and optimal dispenser densities, reflecting landscape heterogeneity (Papaïx et al. 2011, Epanchin-Niell et al. 2012, Potamitis et al. 2015, Schieler et al. 2024, Brewer and Dorman 2025). Economic optimization balances deployment cost and expected yield losses (Mumford and Norton 1984, Lichtenberg and Zilberman 1986). Real-world systems, including FAO eLocust, USDA monitoring networks, and commercial smart traps, integrate sensing, data transmission, and predictive analytics to support regional pest management. Robust data integration and standardization remain critical to handle heterogeneous sources, missing data, and sensor variability.

#### Challenges and Future Directions

Operational implementation faces multiple constraints. Long-term, high-resolution datasets are limited, and environmental sensor coverage remains sparse. Model transferability across regions is challenging due to climate, pest populations, and management differences. Transfer learning may help but requires local calibration (Hossen et al. 2025). Prospective validation is essential, as retrospective datasets can overestimate performance. Data interoperability, standardization, and robust pipelines are necessary. Economic constraints limit high-resolution monitoring, real-time analytics, and automated deployment. Human-in-the-loop integration is crucial for usability and adoption. Environmental and climate variability, sensor reliability, and maintenance complicate operations. Ethical and regulatory considerations, including data privacy and governance, must be addressed. Integrating spatial optimization, timing, dosage, and predictive modeling enables precision deployment. Hybrid mechanistic–data-driven approaches capture complex patterns while retaining interpretability. Future research should explore multispecies models, AI-based anomaly detection, and hybrid approaches for under-sampled regions, while continuously evaluating economic and ecological trade-offs to achieve sustainable deployment.

## Sensor-Based Early Warning and Real-Time Monitoring

Real-time detection of plant volatile emissions provides early warning of pest presence or plant stress before visual symptoms appear. VOCs change predictably when plants are attacked or stressed, and sensor systems can capture these changes. Sensor-based early warning combines chemical sensing with ML to generate actionable risk information, supporting proactive pest management and aligning with the Detection stage of the IPM framework (Cui et al. 2018, Rabehi et al. 2024).

### Volatile Sensing Technologies

Field-ready VOC sensors must balance sensitivity, selectivity, portability, and robustness, yet no single technology meets all requirements. Electronic noses (e-noses) employ arrays of broadly responsive sensors to generate VOC “fingerprints,” distinguishing complex gas mixtures. They are more portable and cost-effective than laboratory instruments, but field performance often declines due to environmental variability (Wilson and Baietto 2009, Zheng and Zhang 2022, Li et al. 2024). Beyond detecting plant stress and herbivore-induced volatiles, e-noses have increasingly been applied to detect insect presence directly through species-specific volatile fingerprints. Studies have demonstrated that sensor arrays coupled with ML classifiers can discriminate stored product pests and identify hidden infestations from insect-emitted volatile blends, highlighting the potential of VOC-based sensing for nondestructive pest surveillance and early warning (Lampson et al. 2014, Chen et al. 2024, Badgujar et al. 2026). Common e-nose sensors include metal oxide semiconductors (MOS), conducting polymers, quartz crystal microbalances, and surface acoustic wave sensors. MOS devices are broadly sensitive but affected by humidity and require heat, while conducting polymers operate at room temperature with tunable selectivity but suffer from drift and shorter lifespans (Bai and Shi 2007, Röck et al. 2008). Portable GC-MS and gas chromatography surface acoustic wave (GC-SAW) systems provide higher chemical specificity, allowing in situ identification of pathogen or herbivory biomarkers, though they are bulkier and more expensive (Beck et al. 2015, Yang et al. 2025). Direct injection mass spectrometers, such as PTR-MS and SIFT-MS, enable real-time quantitative monitoring without chromatographic separation, capturing low-concentration volatiles and diel variability, but high cost and operational complexity limit field deployment (Smith and Spanel 2005, Lindwall et al. 2015). Optical and spectroscopic sensors detect specific marker volatiles with high sensitivity, suitable for targeted monitoring rather than broad VOC profiling (Jiao et al. 2019, Pathak and Viphavakit 2022). Across all platforms, baseline drift, temperature and humidity effects, and background VOC interference can reduce detection reliability unless addressed through calibration, environmental compensation, and adaptive sampling (Hong et al. 2023, Sun and Zheng 2023, Wörner et al. 2025, Lin and Zhan 2026).

### ML for Early Detection

ML extracts meaningful patterns from complex VOC outputs, enabling detection of pest presence, infestation severity, and plant stress (Cui et al. 2018, Herrmann et al. 2024). Supervised algorithms include RF, SVMs, and neural networks. RF handles correlated features and mixed data types while providing variable importance measures (Ranganathan and Borges 2010). SVMs perform well for binary and multiclass detection (Jaleel et al. 2023), while deep learning approaches, such as CNNs, capture temporal patterns in dynamic sensor data but require large datasets (Han et al. 2019, Mei et al. 2025). Effective modeling requires preprocessing, including baseline correction, normalization, drift compensation, and dimensionality reduction, to mitigate environmental noise and sensor ageing (Yan et al. 2015, Fernandez et al. 2025). Models can go beyond presence–absence detection to estimate infestation severity, distinguish pest species, and integrate temporal and spatial data to identify hotspots (Marković et al. 2021, Lee and Yun 2023, Venkateswara and Padmanabhan 2025). Evaluation using accuracy, F1-score, AUC, and Root Mean Square Error is critical, particularly for imbalanced datasets, and methods such as data augmentation or synthetic VOC generation enhance generalization (Chawla et al. 2002, Mujahid et al. 2024). Interpretability tools like SHAP and feature importance analysis reveal influential sensors or compounds, supporting actionable insights (Ko et al. 2025, Mohammad et al. 2025, Ansari et al. 2026). Field performance can decline due to temperature, humidity, background VOCs, and sensor drift, requiring recalibration, adaptive retraining, or transfer learning to maintain reliability (Ma et al. 2018, Yan 2026). Linking model outputs to decision thresholds translates complex VOC patterns into practical pest management guidance (Liakos et al. 2018, Domingues et al. 2022, Amiri and Bandani 2026).

### Integrating Chemical Signals into Decision Support

DSS maximize sensor value by integrating real-time VOC data with spatial, environmental, and predictive models to generate risk alerts and recommend interventions aligned with IPM practices (McBratney et al. 2005, Liakos et al. 2018). Combining sensor outputs with field history, crop phenology, weather, and spatial layers improves detection reliability and contextualizes risk, while multisource fusion further enhances accuracy (Khaki and Wang 2019, Zhang et al. 2019). DSS should quantify uncertainty through confidence scores, probabilities, and potential false alarm costs to aid decision-making (Khosravi et al. 2011). Real-time alerts and dashboards designed for growers enable rapid response, clear communication, confidence, recommended actions, and expected outcomes (Rose et al. 2016). Platforms may run on cloud infrastructure for large-scale integration and model updates, on edge devices for low-latency inference, or hybrid systems balancing responsiveness and scalability (Shi et al. 2016, Wolfert et al. 2017). Performance improves through feedback loops incorporating intervention outcomes, although adaptive learning currently complements periodic recalibration (Liang et al. 2021, Sun et al. 2025). Explainable ML helps identify VOC features contributing to detections and supports biological interpretation, even when overlapping signals prevent direct attribution (Ribeiro et al. 2016, Trilles et al. 2020). Most systems are still validated in controlled or semi-controlled conditions, emphasizing the need for standardized metrics capturing detection latency, false alarms, and economic outcomes (Vergara et al. 2012). Practical deployment also depends on interoperability with farm management systems, cost, maintenance, and regulatory alignment (Hameed et al. 2025, Sarker et al. 2025). Finally, DSS must account for temporal dynamics, linking detection signals to optimal intervention windows, and human factors, ensuring recommendations are clear and aligned with user experience for long-term adoption and trust under real agricultural conditions (Pierpaoli et al. 2013, Gutiérrez et al. 2019, Kendall et al. 2022, Yeo and Keske 2024).

## Explainable ML and Decision Support

**Fig. 6.**
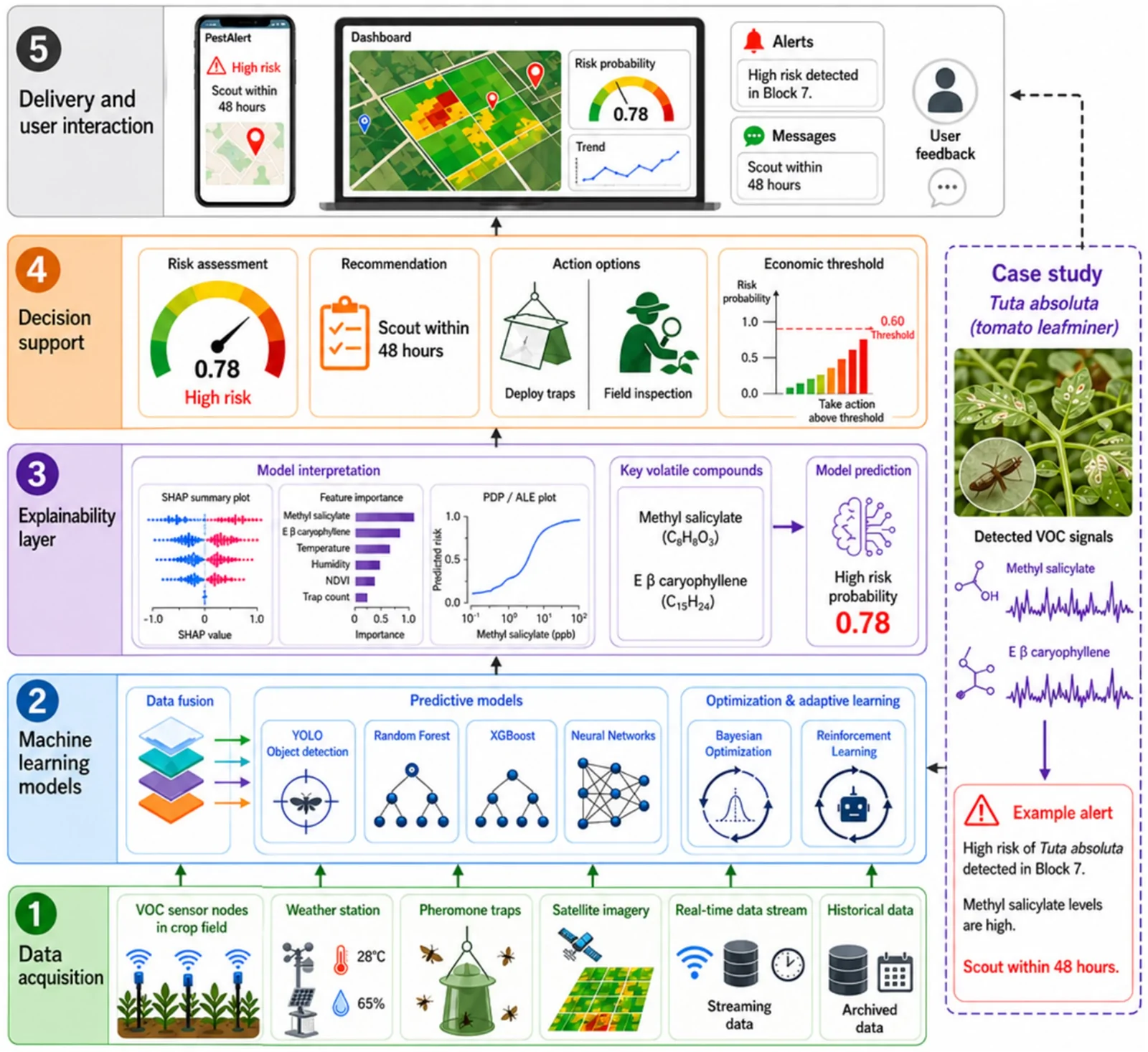
Conceptual framework for integrating explainable machine learning into pest management decision support systems. The figure presents a 5-layer architecture linking data acquisition to field-level action. Solid arrows indicate the flow of information, while a dashed feedback loop supports continuous model refinement. “Layer 1” integrates multisource inputs, including VOC sensors, weather stations, pheromone traps, and satellite imagery, capturing real-time, environmental, and spatial variability. “Layer 2” applies machine learning models for detection, prediction, and optimization, combining object detection, ensemble methods, and adaptive optimization across heterogeneous data streams. “Layer 3” provides explainability using feature importance, SHAP, LIME, and response curves to identify key drivers and link predictions to specific volatile compounds. “Layer 4” translates output into decision support products, including risk alerts, key drivers, and actionable recommendations aligned with economic thresholds. “Layer 5” delivers outputs through user interfaces such as mobile alerts and dashboards, while user feedback enables adaptive learning. A side panel illustrates a *Tuta absoluta* case study in tomato, linking detected volatile signals to real-time alerts and recommended scouting actions. The framework integrates detection, prediction, explanation, and decision-making within a unified system and highlights the need for validation across diverse field conditions.

ML predictions alone are insufficient for pest management. Users must understand recommendations to act on them, and regulators require transparency. In chemical ecology, interpretability reveals which volatiles and interactions influence pest behavior. Explainable ML links chemical signals to actionable insights and integrates Detection, Design, and Deployment into operational Decision Support. Figure 6 presents a conceptual architecture for such integration; while individual components have been validated in isolation, the complete integrated pipeline remains an open research challenge.

### Interpretable Models Linking Chemical Signals to Behavior

Chemical ecology data are complex, with high-dimensional volatile profiles, nonlinear responses, and context-dependent interactions. Sophisticated ML models such as deep neural networks, gradient-boosting ensembles, and nonlinear SVM achieve high predictive accuracy but obscure biological mechanisms (Hassija et al. 2024, Mehdiyev et al. 2025). Interpretability validates model reliability and generates testable hypotheses. Intrinsically interpretable models, linear, logistic, decision tree, and rule-based models, produce outputs traceable to inputs, allowing identification of volatiles driving predictions. For example, Baleba et al. (2019) identified β-citronellene and carvone as oviposition predictors in *S. calcitrans*, while Peterson et al. (2020) reduced 59 host plant volatiles to 8 discriminants for testing against *A. planipennis.* sPLS balances complexity and interpretability (Lê Cao et al. 2011); Ayelo et al. (2022) combined sPLS with RF to reveal α-pinene, β-myrcene, methyl salicylate, and *(E)-*β-caryophyllene as attractants for *D. gelechiidivoris*. Simple models may underfit, as insect responses to plant volatiles are rarely linear and often involve synergistic or antagonistic effects (Conchou et al. 2019).

### Post-hoc Explainable AI Methods

Post-hoc methods approximate complex model behavior without reducing predictive power. Feature importance techniques, including permutation importance and SHAP, quantify contributions (Ribeiro et al. 2016). López-Cortés et al. (2025) employed XGBoost to predict volatile–odorant protein binding affinities. Partial dependence plots (PDPs) and individual conditional expectation curves visualize feature effects (Goldstein et al. 2015), while accumulated local effects (ALE) plots account for correlated volatiles (Apley and Zhu 2020). Local methods such as LIME generate feature contributions for individual predictions, supporting field verification (Ribeiro et al. 2016, Kenny and Huang 2022). Visualization-based methods, including attention mechanisms and saliency maps, reveal features driving neural network predictions; Nguyen et al. (2026) used UMAP to visualize mosquito olfactory neural responses and guide compound prioritization. Interpretability drives biological discovery: RF feature importance identified monoterpenes as attractants and sesquiterpenes as repellents in tomato tri-trophic interactions (Adams et al. 2023), and distinguished phenol and indole as oviposition deterrents in *Drosophila* fecal volatiles (Mansourian et al. 2016).

### Enhancing Practitioner Confidence and Adoption

Adoption of ML-based decision support depends on practitioner trust. Users rarely act on recommendations they do not understand, especially when interventions carry economic or environmental costs (Rose et al. 2016, Lindblom et al. 2017). Explainable outputs align predictions with field observations, making them credible. Sensor-based alerts highlight elevated pheromone-like compounds in zones consistent with pest phenology. Visualizations and plain-language summaries increase trust (Kenny and Huang 2022). For example: Alert: “Elevated (E)-β-caryophyllene and methyl salicylate detected” (Miano et al. 2022). Elevated risk of *T. absoluta* infestation predicted. Consider scouting within 48 h. Such alerts can be generated by integrating object detection models (eg YOLO) for pest identification with large language models (LLMs) for natural language generation, an approach that has shown promise in reducing crop damage in field settings (Şahin et al. 2025). Human-in-the-loop frameworks allow practitioners to flag false positives or negatives, supporting model retraining (Settles 2009, Domingues et al. 2022). Explainable outputs also provide auditability for IPM compliance and regulatory transparency under standards such as the General Data Protection Regulation (GDPR) (Goodman and Flaxman 2017). In low-resource settings, accessible outputs like color-coded maps or simple alerts enable smallholder farmers to act independently, as demonstrated in the Innovate UK bioacoustic palm weevil monitoring project in Ghana (Innovate UK 2025). Barriers include a lack of standardized explainability metrics, limited user-centerd design, and variable technical literacy, highlighting the need for collaborative development among ML researchers, chemical ecologists, social scientists, and end-users (Rose et al. 2016).

#### Operational Framework for IPM Decisions

ML-enabled chemical ecology adds value when integrated into decision systems, linking Detection, Design, Deployment, and Decision Support. Sensor arrays generate early warning signals, and explainable classifiers identify key compounds. Predictive models guide semiochemical design, with feature importance informing blend optimization and behavioral testing. Optimization models direct spatial and temporal deployment, highlighting priority zones and timing rationale. Decision support integrates these outputs into interpretable guidance for validation and adaptive learning. Explainable outputs communicate infestation probabilities, key drivers, and uncertainties, enabling interventions aligned with Economic Injury Levels and Economic Thresholds (Pedigo et al. 1986, Hutchins et al. 1988) and supporting risk-based decisions (van den Bosch and Gilligan 2008). The workflow combines volatile sensors, environmental monitors, trap counts, phenology models, and spatial data with ML models, explainability layers (SHAP, LIME, PDP, ALE, feature importance), and decision layers integrating thresholds, maps, and economic analysis. Practitioner feedback supports iterative refinement. Evaluation metrics include predictive accuracy, user adoption, decision quality, economic outcomes, and pest suppression, with prospective field validation ensuring robustness (Domingues et al. 2022). Future directions include standardizing explainability reporting, applying user-centerd design, integrating LLMs for adaptive explanations, assessing effects on trust and decision quality, and developing modular open-source frameworks for diverse cropping systems.

## Challenges, Data Integration, and Recommendations

ML can transform chemical ecology for integrated pest management, but progress depends on addressing limitations in data availability, methodological standards, and geographical coverage. Key constraints include small and fragmented datasets, limited shared benchmarks, restricted field validation, and a bias toward well-resourced temperate systems (van Klompenburg et al. 2020, Sharma et al. 2023). This section outlines practical steps to build reusable datasets and develop ML-enabled IPM tools applicable across agricultural contexts, with particular focus on tropical agroecosystems and smallholder systems where sustainable alternatives to broad-spectrum insecticides are needed.

### Data Scarcity, Heterogeneity, and Benchmarking

Progress is limited by the lack of large, well-annotated datasets. Data are complex: GC-MS profiles can contain hundreds of compounds, behavioral assays generate binary, count, or trajectory outputs, electrophysiology produces temporally structured signals, and field studies add spatial and temporal variation. Sample sizes are often small (3 to 10 replicates per treatment), supporting standard statistical testing but constraining ML model training, especially for deep learning approaches that require larger datasets (Lu et al. 2020, Liu and Wang 2021). Heterogeneity adds further constraints: volatile profiles originate from GC-MS, PTR-MS, SIFT-MS, and electronic noses, each with different sensitivity and coverage, and compound annotation varies in quality (Xin et al. 2018, Kan et al. 2025, Katherinatama et al. 2025). Variability in VOCs collection methods and batch effects on GC-MS datasets further complicate cross-study comparisons. Behavioral assays follow diverse protocols, electrophysiological data differ in sampling and preprocessing, and environmental data are inconsistently recorded (Mundinger et al. 2025). These differences limit integration and aggregation. Unlike fields such as computer vision, chemical ecology lacks benchmark datasets with standardized splits, making method comparison challenging. Open repositories, standard metadata, and routine field validation across seasons and locations are needed to improve reproducibility and utility.

### Reusable and Interoperable Dataset Construction

Building ML-ready data infrastructure requires deliberate design for reusability and interoperability. Linking multimodal data is essential, as single studies often generate behavioral trajectories, chemical profiles, electrophysiology, and spatiotemporal field data that are frequently stored in isolation. Data should be connected using consistent sample identifiers, shared metadata, and standardized formats so that volatile profiles can be associated with behavioral responses, electrophysiological signals, and environmental conditions, enabling models to learn across data types. Chemical annotation should use machine-readable identifiers such as the IUPAC International Chemical Identifier keys, PubChem Compound Identifiers, or Simplified Molecular Input Line Entry System, supporting integration with molecular databases and enabling QSAR modeling and virtual screening (Rogers and Hahn 2010). Spectral libraries should be shared in open formats, and sensor data should be archived with preprocessing details. Behavioral data require clear response definitions, documented protocols, and archiving of raw trajectory coordinates alongside derived metrics to facilitate reuse. Environmental and spatial metadata should include temperature, humidity, wind, georeferenced field locations, and crop phenology aligned with other measurements, allowing models to capture context and generalize across locations. Open repositories such as National Center for Biotechnology Information, Knowledge Network for Biocomplexity, and Pherobase should be used, following Findable, Accessible, Interoperable, and Reusable (FAIR) principles for findability, accessibility, interoperability, and reusability, with accompanying code for reproducibility (Wilkinson et al. 2016). The development of benchmark datasets for priority tasks—eg volatile-based pest detection or receptor-ligand prediction with standardized splits and metrics—would enable rigorous comparison of methods and accelerate field-wide progress.

### Special Considerations for Tropical Agroecosystems and Smallholder Systems

Most ML-enabled chemical ecology studies focus on well-resourced temperate systems, leaving tropical agroecosystems and smallholder systems underrepresented despite high pest pressure and limited access to chemical inputs. Tropical systems differ ecologically: year-round pest pressure, overlapping generations, greater crop diversity, and environmental conditions such as high temperature and humidity influence sensor performance and semiochemical dynamics. Additionally, the high diversity of understudied and invasive insect species, together with sparse reference data, complicates modeling. Infrastructure constraints in smallholder systems, variable connectivity, intermittent electricity, and limited access to digital tools require low-cost, practical solutions that leverage local resources. Addressing these constraints involves developing affordable sensor platforms (MOS-based electronic noses or simple colorimetric tools), models that perform with limited data using transfer learning, few-shot learning, or active learning, and interpretable mobile tools designed for offline use. Capacity building and equitable collaboration are critical: researchers in tropical regions should gain ML expertise, while ML researchers engage with field realities, supported by shared leadership, data ownership, and training initiatives. Finally, tools must be validated under real-world conditions across seasons, pest pressures, and management practices, while evaluating both technical performance and economic outcomes to ensure adoption and practical impact.

## Conclusion

### Applied Framework Summary

ML is a robust approach that can transform the study and management of insect–plant interactions by complementing traditional statistical methods. High-dimensional volatile profiles, nonlinear behavioral responses, temporally structured electrophysiology, and spatiotemporally autocorrelated field data are challenging for conventional models. ML can detect patterns in unlabeled data, generate candidate molecules, and optimize interventions across space and time. This review underscored an integrated framework with 4 stages: Detection, Design, Deployment, and Decision Support. Detection uses sensor-based systems with classifiers to identify pest presence or plant stress before visible symptoms. Design employs predictive and generative models to prioritize compounds and optimize blends. Deployment integrates landscape, phenology, pest dynamics, and environmental variability to guide application timing and location. Decision Support uses explainable models to translate outputs into actionable recommendations, supporting adaptive IPM decisions. Integration across stages connects chemical discovery to field-scale implementation, enhances precision, supports biological control, reduces reliance on broad-spectrum insecticides, and enables adaptive responses.

### Future Directions for ML in Chemical Ecology

Despite the numerous advantages offered by ML in chemical ecology, several challenges remain. These include data scarcity, dataset fragmentation and heterogeneity, the absence of shared benchmark datasets, limited validation under field conditions, and the underrepresentation of tropical and smallholder production systems. Addressing these gaps requires end-to-end pipelines linking detection, design, deployment, and decision support, where sensor-based systems inform semiochemical cue identification or optimization, and outputs feed into interpretable tools. Generative models should be linked to predictive screening, synthesis, electrophysiology, and behavioral assays, although no current study has completed this full workflow. Hybrid mechanistic–ML models offer improved interpretability and generalization by combining biological knowledge with data-driven methods. Real-time adaptive management using low-cost sensors, edge computing, and reinforcement learning can replace static schedules with responsive interventions. Future models should capture multispecies and community-level interactions, balancing pest suppression with conservation of beneficial species. Expanding chemical space via ML allows the design of semiochemicals with properties tailored to field conditions, incorporating toxicity and environmental fate. Equitable solutions require low-cost technologies, models effective with limited data, participatory design, and capacity building. Finally, standardization and benchmarking using FAIR principles, open repositories, and community-agreed metrics are essential to ensure reproducibility, comparison, and cumulative progress, supported by policy incentives from journals and funders.

## Data Availability

No new data were generated or analyzed in this review. All information is derived from previously published studies cited in the references.
